# Accelerated Degradation of Poly-ε-caprolactone Composite Scaffolds for Large Bone Defects

**DOI:** 10.3390/polym15030670

**Published:** 2023-01-28

**Authors:** Evangelos Daskalakis, Mohamed H. Hassan, Abdalla M. Omar, Anil A. Acar, Ali Fallah, Glen Cooper, Andrew Weightman, Gordon Blunn, Bahattin Koc, Paulo Bartolo

**Affiliations:** 1 School of Mechanical, Aerospace and Civil Engineering, University of Manchester, Manchester M13 9PL, UK; 2 Integrated Manufacturing Technologies Research and Application Center, Sabanci University, Tuzla 34956, Istanbul, Turkey; 3 SUNUM Nanotechnology Research Center, Sabanci University, Tuzla 34956, Istanbul, Turkey; 4 Faculty of Engineering and Natural Sciences, Sabanci University, Tuzla 34956, Istanbul, Turkey; 5 School of Pharmacy and Biomedical Sciences, University of Portsmouth, Portsmouth PO1 2DT, UK; 6 Singapore Centre for 3D Printing, School of Mechanical and Aerospace Engineering, Nanyang Technological University, Singapore 639798, Singapore

**Keywords:** 3D printing, additive manufacturing, biomaterials, degradation process

## Abstract

This research investigates the accelerated hydrolytic degradation process of both anatomically designed bone scaffolds with a pore size gradient and a rectangular shape (biomimetically designed scaffolds or bone bricks). The effect of material composition is investigated considering poly-ε-caprolactone (PCL) as the main scaffold material, reinforced with ceramics such as hydroxyapatite (HA), β-tricalcium phosphate (TCP) and bioglass at a concentration of 20 wt%. In the case of rectangular scaffolds, the effect of pore size (200 μm, 300 μm and 500 μm) is also investigated. The degradation process (accelerated degradation) was investigated during a period of 5 days in a sodium hydroxide (NaOH) medium. Degraded bone bricks and rectangular scaffolds were measured each day to evaluate the weight loss of the samples, which were also morphologically, thermally, chemically and mechanically assessed. The results show that the PCL/bioglass bone brick scaffolds exhibited faster degradation kinetics in comparison with the PCL, PCL/HA and PCL/TCP bone bricks. Furthermore, the degradation kinetics of rectangular scaffolds increased by increasing the pore size from 500 μm to 200 μm. The results also indicate that, for the same material composition, bone bricks degrade slower compared with rectangular scaffolds. The scanning electron microscopy (SEM) images show that the degradation process was faster on the external regions of the bone brick scaffolds (600 μm pore size) compared with the internal regions (200 μm pore size). The thermal gravimetric analysis (TGA) results show that the ceramic concentration remained constant throughout the degradation process, while differential scanning calorimetry (DSC) results show that all scaffolds exhibited a reduction in crystallinity (Xc), enthalpy (Δm) and melting temperature (Tm) throughout the degradation process, while the glass transition temperature (Tg) slightly increased. Finally, the compression results show that the mechanical properties decreased during the degradation process, with PCL/bioglass bone bricks and rectangular scaffolds presenting higher mechanical properties with the same design in comparison with the other materials.

## 1. Introduction

A major concern related to the design of polymer-based bone tissue engineering scaffolds is related to the degradation process and degradation kinetics of these constructs as well as the rate of tissue regeneration. After generating an in vitro cell culture of a scaffold/tissue system, the degree of remodelling and replacement of the biological implant by the native tissue needs to be considered [1,2,3,4,5,6,7]. Tissue remodelling is important to obtain stable mechanical conditions and vascularization at the implantation site [8,9,10,11,12,13,14,15]. Therefore, scaffolds should maintain sufficient structural integrity during the tissue regeneration process, which must be accompanied by a similar scaffold’s degradation kinetics [16,17,18,19,20].

In the case of synthetic polymers such as poly-ε-caprolactone (PCL), hydrolysis is considered the most important degradation mechanism, which strongly depends on the type of chemical bonds, copolymer composition and wettability properties [21,22,23,24,25]. This degradation mechanism involves the hydrolysis of unstable ester bonds present in the molecular chain of the polymer [26,27,28,29,30]. Moreover, as these polymers are semi-crystalline materials, it has been pointed out that the degradation occurs through a random hydrolytic split of the amorphous regions followed by the gradual degradation of the crystalline regions [31,32,33,34,35,36,37,38]. However, the degradation mechanism depends on multiple factors related to both the material (e.g., crystallinity and molecular weight) and topological (e.g., pore size and pore shape) characteristics of scaffolds, which are not fully understood. Several papers have been published based on rectangular or cylindrical scaffolds, investigating the effect of pore size and pore architecture and material composition [39,40,41,42,43,44,45,46,47]. However, a comparison of the degradation kinetics of polymer/ceramic scaffolds containing different ceramic materials (e.g., hydroxyapatite (HA), β-tricalcium phosphate (TCP) and bioglass) is still missing.

Despite the significant advances in the field of bone tissue engineering, one of the biggest challenges from a scaffold design point of view is the difficulty in producing structures capable of simultaneously presenting (1) high mechanical strength and (2) proper degradation kinetics.

Through the design of anatomical design scaffolds with gradient pore sizes mimicking the structure of bone (bone bricks), it was possible to achieve mechanical properties suitable for bone engineering applications, which represents a significant step forward in the field [48,49]. This research investigates the accelerated hydrolytic degradation process of bone bricks with different material compositions to fully understand the effects of gradient pore size and material content on the behaviour of bone brick scaffolds. Accelerated degradation was considered as an alternative to the use of phosphate-buffered saline (PBS) or simulated body fluid due to the long-term degradation of PCL (2–3 years) in physiological conditions, providing relevant data on the behaviour of the different considered materials [50]. This allowed us to investigate, for the first time, the behaviour of PCL-based scaffolds reinforced with the most used ceramic materials. Moreover, the degradation kinetics are compared for rectangular scaffolds with different pore sizes and similar material compositions, providing relevant information on the impact of scaffold architecture.

## 2. Materials and Methods

### 2.1. Materials

PCL (melting point = 60 °C, glass transition temperature= −60 °C and molecular weight = 50,000 Da; CAPA 6500, Perstorp Caprolactones, Cheshire, UK) was used as received in the form of pellets. Composite blends were prepared using HA (molecular weight = 502.31 g/mol and melting point = 1100 °C; Sigma-Aldrich, St. Louis, MO, USA) in nanopowder shape (<20 nm particle size), TCP (molecular weight = 310.18 g/mol and melting point = 1391 °C; Sigma-Aldrich, St. Louis, MO, USA) in powder shape (ranging between 20 μm and 30 μm) and bioglass 45S5 (6 wt% P_2_O_5_, 45 wt% SiO_2_, 24.5 wt% Na_2_O and 24.5 wt% CaO; CeraDynamics Ltd., James Kent Group, Stoke, UK) in powder shape (<10 μm particles size). A melt blending process was used to prepare the blends.

### 2.2. Bone Bricks and Scaffolds Production

**Figure 1:**
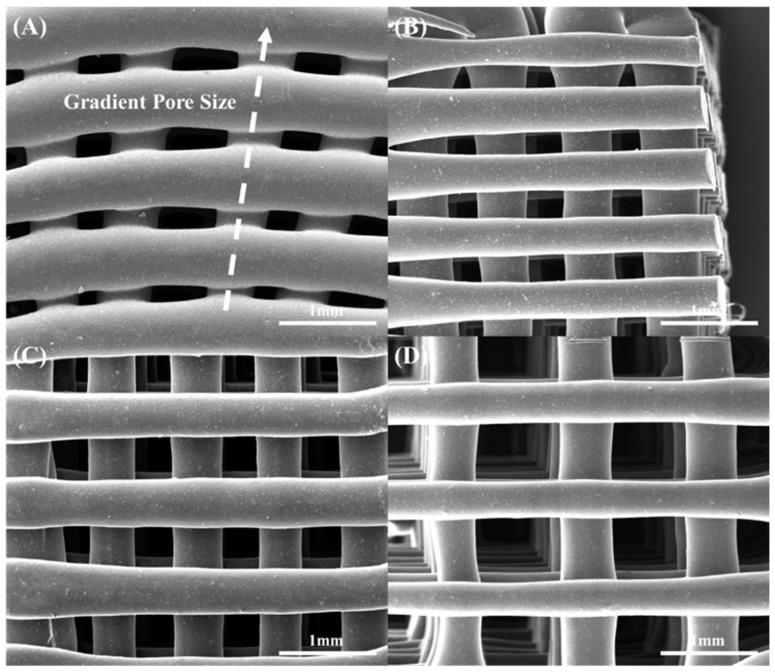
SEM images of (**A**) bone bricks (PCL/bioglass), (**B**) 200 μm pore size rectangular scaffolds (PCL/bioglass), (**C**) 300 μm pore size rectangular scaffolds (PCL/bioglass) and (**D**) 500 μm pore size rectangular scaffolds (PCL/bioglass).

Bone bricks with different material compositions (PCL, PCL containing 20 wt% of HA, PCL containing 20 wt% of TCP, and PCL containing 20 wt% of bioglass), were fabricated using the screw-assisted additive manufacturing 3D Discovery (RegenHU, Villaz-St-Pierre, Switzerland) and a continuous path algorithm based on 38 zig-zag double filaments and 14 spiral filaments (Figure 1). Similar material compositions were considered for the fabrication of rectangular scaffolds with uniform pore sizes. A total of 3 different pore sizes were considered: 200 μm, 300 μm and 500 μm (Figure 1). The overall dimensions of the bone bricks were 31 mm × 26.7 mm × 10 mm. Both bone bricks and rectangular scaffolds were printed using the following processing parameters: 90 °C melting temperature, 20 mm/s deposition velocity and 12 rpm screw rotational velocity. The filaments were extruded using a 0.33 mm diameter needle.

### 2.3. Degradation Procedure

Accelerated degradation studies were conducted using sodium hydroxide (NaOH) of 5 mol/L (5 N) in aqueous solution (VWR, Radnor, PA, USA) with a density of 1.185 gr/cm^3^ (20 °C), a solubility of 20 °C and a pH of 14 (H_2_O, 20 °C). The degradation period took place for 5 days. On each day, 5 samples were used from each considered case and measured using a high-precision balance. At each time point, the samples were removed from the NaOH and washed three times with the use of deionised water and left to dry overnight. Once completely dry, the samples were measured to determine the weight reduction. The amount of NaOH used for 5 rectangular scaffolds was 15 mL and for the 5 anatomically designed bone bricks, the amount was 50 mL (due to their size). The pH was monitored throughout the experimental work, and no changes were observed (pH of 14).

### 2.4. Morphological Characterization

Scanning electron microscopy (SEM) was used to investigate the morphological characteristics of the samples and to determine pore sizes (FEI ESEM Quanta 250, FEI Company, Hillsboro, OR, USA). Scaffolds were coated (platinum coating) with the use of an EMITECH K550X sputter coater (Quorum Technologies, Laughton, East Sussex, UK) before imaging. The SEM images were analysed using ImageJ 1.x (National Institutes of Health, Bethesda, MD, USA) (10 measurements per sample).

The porosity of the scaffolds was calculated with the use of the following equation:

(1) $$P_{t}=\left(\frac{V_{p}}{V}\right)\times 100$$

where *V_p_* is the pore volume, *V* is the total bulk volume and *P_t_* is the porosity.

The density of PCL used in these experiments was calculated as follows:

(2) $$\rho =\frac{m}{V}$$

where *ρ* is the density of the unprocessed material, m is the mass measured and *V* is the volume measured. The calculated density of PCL was 1.124 ± 0.003 g/cm^3^.

### 2.5. Thermal Gravimetric Analysis

Thermal gravimetric analysis, using a TGA Q500 (TA Instruments, New Castle, UK), was used to investigate the thermal degradation and to calculate the ceramic content on the printed scaffolds. Experiments were repeated 4 times per considered scaffold (n = 4). The tests were conducted in air atmosphere (50 mL/min) with a temperature ranging from 25 °C to 1000 °C at a rate of 10 °C/min. The weight of each sample was 200 mg, and each test was conducted twice.

### 2.6. Differential Scanning Calorimetry

Differential scanning calorimetry (DSC) tests were performed to determine the melting temperature (Tm), the enthalpy (ΔHm), the crystallinity and the glass transition temperature (Tg) (n = 4). Tests were conducted using a TA Q100 (TA Instruments, New Castle, UK) under a nitrogen/air atmosphere (50 mL/min). The heating cycle was as follows: heating from −90 °C to 100 °C at a rate of 10 °C/min and then keeping stable for 2 min. The weight of each considered sample was 20 mg.

### 2.7. Mechanical Testing

Compression tests were performed using an INSTRON 3344 (Instron, High Wycombe, Buckinghamshire, UK) (n = 4), at different degradation time points, according to the ASTM D695-15. Force versus displacement curves obtained using the Bluehill Universal Software (Instron, High Wycombe, Buckinghamshire, UK) were stress–strain curves. The compressive modulus was determined based on the slope of the elastic region of the stress–strain curves.

### 2.8. Data Analysis

Statistical analysis was performed using a one-way analysis of variance (one-way ANOVA) with Tukey’s post hoc test (GraphPad Software Inc., San Diego, CA, USA). Weight loss, TGA and DSC data were analysed using Origin 2021 (Origin Lab Corporation, Northampton, MA, USA) and are presented as average values of the obtained results.

## 3. Results and Discussion

### 3.1. Degradation Analysis

**Figure 2:**
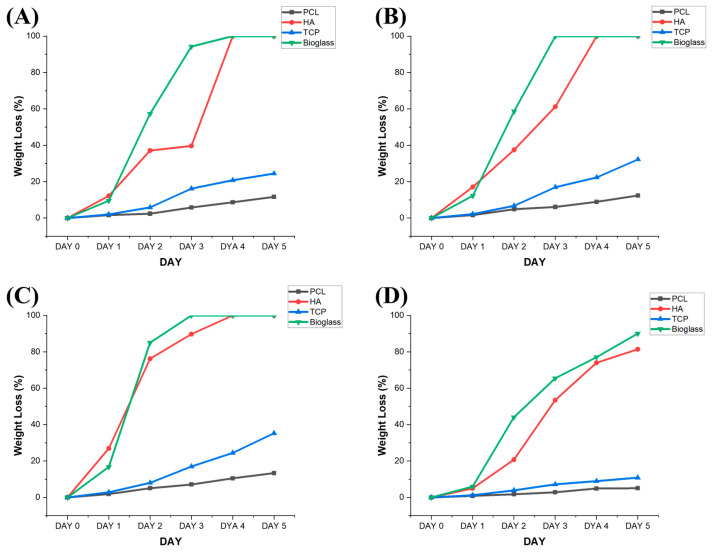
Weight loss as a function of degradation time for different samples and material compositions. (**A**) 200 μm pore size rectangular scaffolds, (**B**) 300 μm pore size rectangular scaffolds, (**C**) 500 μm pore size rectangular scaffolds and (**D**) bone bricks.

Figure 2 shows the weight reduction values for the different samples at different degradation time points. The results show that the addition of ceramic materials accelerates the degradation process, with the PCL bone bricks exhibiting the lowest weight reduction at day 5 (5.07%) and PCL/bioglass the highest weight loss (90%) followed by PCL/HA (81.41%) and PCL/TCP (10.85%). In the case of rectangular scaffolds, the results show that PCL/bioglass samples also degrade faster than their PCL/HA and PCL/TCP counterparts. Moreover, it was possible to observe that the increase in pore size accelerated the degradation process. The PCL/bioglass scaffolds with a 200 μm pore size were fully degraded after day 4, while the scaffolds with 300 μm and 500 μm pore sizes degraded after day 3. For PCL/HA, the results show that all samples (200 μm, 300 μm and 500 μm) were fully degraded after day 4. In the case of the PCL/TCP scaffolds, which were not fully degraded after day 5, the weight loss increased from 24.45% for scaffolds with a 200 μm pore size to 35.25% for scaffolds with a 500 μm pore size. A similar trend was observed for the PCL scaffolds (showing an 11.64% weight loss at day 5 for scaffolds with a 200 μm pore size and a 13.37% weight loss at day 5 for scaffolds with a 500 μm pore size), which exhibited the lowest degradation kinetics. This can be explained by the increase in the surface area exposed to the NaOH that accelerates the hydrolytic degradation and the release into the liquid medium of the ceramic particles previously bonded with the polymeric material [51,52,53,54,55,56,57,58]. Results also indicate that, for the same material composition, bone bricks degrade slower compared with rectangular scaffolds. As the overall porosity of the bone bricks is 52%, while the porosity of the rectangular scaffolds varies between 42% (scaffolds with a 200 μm pore size) and 56% (scaffolds with a 500 μm pore size), the observed differences can be attributed to the pore size gradient created in the bone bricks, which superimposes both the porosity and overall pore size (with an average of 350 μm in the bone bricks).

The effects of the scaffold architecture and material composition on the degradation kinetics can also be observed in Figures S1–S15 showing SEM images that, for the different scaffolds and degradation time points, illustrate the surface erosion, decrease in the filament diameter and consequent increase in pore size, and filament collapse. Furthermore, from Table 1, Table 2, Table 3 and Table 4 it is possible to observe that both pore size and porosity increase during the 5 days of degradation.

### 3.2. Thermal Gravimetric Analysis

The thermal gravimetric analysis (TGA) results show that all considered samples exhibit degradation temperatures between 304.12 °C and 437.11 °C, after which only the inorganic materials remain (Figures S16 and S17). Moreover, the addition of ceramic particles into the polymer matrix reduces the degradation temperature, which can be observed for all samples throughout the degradation period. Moreover, the results in Table 5 and Figures S16 and S17 show that the overall weight ratio between polymer and ceramic material remains almost constant, while the total amount of ceramic materials significantly decreases, indicating a significant loss of polymer. For example, at day 3, the amount of HA in the samples is 22.4 wt%, and the total amount of HA is 9.4% of the initial HA content, while for the PCL/bioglass samples, the amount of bioglass in the samples is 22.1 wt%, and the total amount of bioglass is 7.1% of the initial bioglass content. These results clearly show a significant loss of polymer in the case of bioglass and confirm the observations discussed in Section 3.2 suggesting that the addition of ceramic materials (bioglass in particular) accelerates the degradation process. Additionally, this behaviour can also be explained by the morphological changes in the polymeric matrix, as discussed in Section 3.2.

As samples were printed at 90 °C, the results show that the printing conditions do not induce any material degradation. Additionally, the levels of HA, TCP and bioglass determined with TGA (Table 1) suggest that the melt blending approach used to prepare the blends is a simple and efficient method.

### 3.3. Chemical Analysis

DSC was used to investigate the crystallinity (Xc), melting temperature (Tm), enthalpy (Δm) and glass transition temperature (Tg) of bone bricks and rectangular scaffolds during the degradation process (Table 6 and Figure S18). The results show that the addition of ceramic materials into the polymer reduces the crystallinity (from 86.91% to 61.08%), the enthalpy (from 89.89 J/g to 65.06 J/g) and the melting temperature (from 64.65 °C to 69.82 °C), while the glass transition temperature slightly increases (from −59.19 °C to −59.1 °C) [58,59,60,61,62,63]. Similar results showing that the addition of ceramic particles constrains the crystallization process, limiting the mobility of PCL chains in the polymer–ceramic matrix and inducing the formation of smaller or thinner regions of crystalline lamellae, were also reported by other groups [64,65,66,67].

The results show that the melting temperature reduced for all the material compositions from day 0 to day 5, with the highest reduction observed for the bioglass scaffolds (there were no significant differences between PCL/bioglass and PCL/HA) and the lowest for TCP scaffolds. This can be explained by the decrease in crystallinity (with the highest reduction in the case of the PCL/HA and PCL/bioglass samples), and the melting temperature can be interpreted as the energy required by a system to destroy ordered regions. The results also show that the degradation occurs mainly through the destruction of the crystalline regions, which is aligned with the variations in both enthalpy and glass transition temperature that decrease and increase during the degradation time, respectively.

### 3.4. Mechanical Analysis

**Figure 3:**
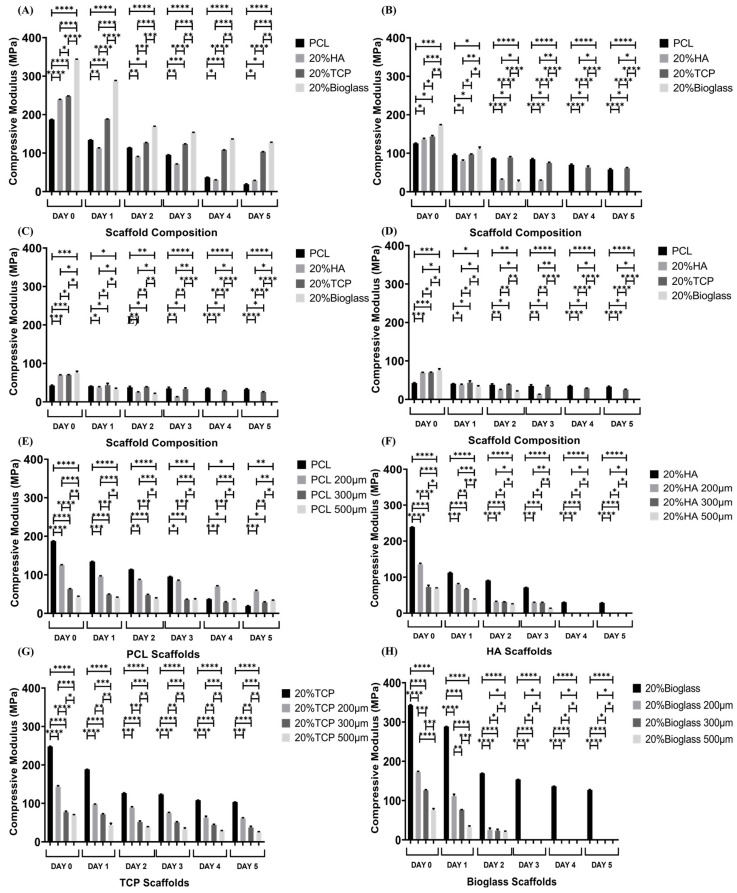
Compressive modulus as a function of degradation time for different architectures and material compositions. (**A**) Bone bricks scaffolds; (**B**) rectangular scaffolds with 200 μm pore size; (**C**) rectangular scaffolds with 300 μm pore size; (**D**) rectangular scaffolds with 500 μm pore size; (**E**) PCL bone bricks and rectangular scaffolds; (**F**) HA bone bricks and rectangular scaffolds; (**G**) TCP bone bricks and rectangular scaffolds; and (**H**) bioglass bone bricks and rectangular scaffolds. * Statistical evidence (*p* < 0.05) analysed by one-way ANOVA, and Tukey post hoc test. The * statistical evidence (*p* < 0.05), **, *** and **** is the one-way analysis of variance (one-way ANOVA) and Tukey’s post hoc test with the use of GraphPad Prism software and is used to show the difference between the results. The * is a small difference, while more * are added as the differences between the results increases.

The compressive modulus results for all considered samples at different degradation time points are indicated in Figure 3. As observed, at day 0 and for the same material composition, the printed bone scaffolds exhibited higher compressive moduli than their rectangular scaffold counterparts, indicating the relevance of the scaffold architecture on the mechanical performance. In the case of the rectangular scaffolds, the results show that for the same material composition, scaffolds with large pore sizes (500 μm) present lower compressive moduli than scaffolds with the lowest pore sizes (200 μm), which can be attributed to the increase in porosity. Furthermore, throughout the degradation process it can be observed that for bone bricks, the PCL/bioglass bone bricks present higher compressive moduli, while for rectangular scaffolds, the PCL/TCP scaffolds present higher compressive properties for all the different pore sizes. Moreover, compressive moduli decreased for all samples throughout the degradation process, which is associated with the decrease in crystallinity, decrease in the filament diameters, increase in porosity, filament collapse and limited adhesion between filaments.

## 4. Conclusions

The degradation kinetics of anatomically designed scaffolds with pore size gradients (bone bricks) and rectangular scaffolds with different pore sizes, considering a range of material compositions, was investigated, taking into consideration weight loss and morphological, thermal, chemical and mechanical changes. Due to the long degradation time of PCL, accelerated degradation, using NaOH, was considered. The results show that bone bricks present faster and more controlled degradation in comparison with rectangular scaffolds. The fastest degradation (weight loss) was observed for PCL/bioglass bone bricks and rectangular scaffolds. Moreover, for the same material composition, the degradation is faster in scaffolds presenting large pore sizes. The TGA results show that, in all considered cases, the concentration of the inorganic material remained the same during the degradation process. The DSC results indicate that the crystallinity of all samples at different degradation times decreased, suggesting a faster destruction of the crystalline regions than the amorphous ones. This observation is contrary to other studies suggesting that the degradation of PCL occurs through an initial mass loss that occurs due to the random hydrolytic split of polymeric chains in the amorphous regions, followed by a gradual degradation in the crystalline regions [63,64,65,66]. High levels of crystalline regions on the surfaces of the printed filaments together with the penetration of the degradation medium promoting internal degradation may explain the obtained results. Finally, as expected, compressive moduli decreased throughout the degradation process. However, for the same material content and degradation time point, bone bricks present better mechanical properties than rectangular scaffolds. Moreover, for the same material composition, better mechanical properties were observed for scaffolds with lower pore sizes. Overall, the accelerated degradation process showed that PCL/bioglass bone brick scaffolds present faster and more controlled degradation kinetics, compared with the other material concentrations and scaffold designs, and higher mechanical properties, making it the most suitable physical support for bone tissue applications.

## Figures and Tables

**Table 1:** Pore size and porosity values of bone brick scaffolds before and after the degradation process.

Materials	Pore Size (μm)	Porosity (%)
**Day 0**		
PCL	333 ± 90	52
HA	336 ± 10	54
TCP	377 ± 64	55
Bioglass	389 ± 47	56
**Day 1**		
PCL	400 ± 56	58
HA	398 ± 31	58
TCP	412 ± 94	60
Bioglass	430 ± 29	62
**Day 2**		
PCL	402 ± 63	58
HA	400 ± 31	58
TCP	420 ± 61	61
Bioglass	599 ± 81	70
**Day 3**		
PCL	428 ± 77	62
HA	657 ± 52	75
TCP	443 ± 26	64
Bioglass	678 ± 41	77
**Day 4**		
PCL	452 ± 42	65
HA	851 ± 69	84
TCP	463 ± 52	66
Bioglass	876 ± 42	86
**Day 5**		
PCL	479 ± 36	67
HA	505 ± 8	90
TCP	510 ± 47	69
Bioglass	616 ± 6	92

**Table 2:** Pore size and porosity values of scaffolds (200 μm pore size) before and after the degradation process.

Materials	Pore Size (μm)	Porosity (%)
**Day 0**		
PCL	205 ± 1	42
HA	207 ± 4	42
TCP	214 ± 3	43
Bioglass	217 ± 8	43
**Day 1**		
PCL	214 ± 5	43
HA	252 ± 3	45
TCP	220 ± 4	43
Bioglass	233 ± 4	44
**Day 2**		
PCL	246 ± 2	45
HA	443 ± 53	64
TCP	249 ± 1	45
Bioglass	333 ± 15	52
**Day 3**		
PCL	249 ± 2	45
HA	505 ± 18	68
TCP	263 ± 4	46
Bioglass	0	100
**Day 4**		
PCL	257 ± 2	45
HA	0	100
TCP	281 ± 6	47
Bioglass	0	100
**Day 5**		
PCL	257 ± 2	45
HA	0	100
TCP	281 ± 6	47
Bioglass	0	100

**Table 3:** Pore size and porosity values of scaffolds (300 μm pore size) before and after the degradation process.

Materials	Pore Size (μm)	Porosity (%)
**Day 0**		
PCL	306 ± 3	50
HA	310 ± 5	50
TCP	312 ± 3	50
Bioglass	315 ± 7	51
**Day 1**		
PCL	314 ± 3	50
HA	394 ± 1	57
TCP	312 ± 3	50
Bioglass	315 ± 7	50
**Day 2**		
PCL	331 ± 2	51
HA	420 ± 3	61
TCP	345 ± 1	53
Bioglass	462 ± 4	66
**Day 3**		
PCL	340 ± 3	52
HA	630 ± 7	73
TCP	354 ± 1	53
Bioglass	0	100
**Day 4**		
PCL	345 ± 2	53
HA	0	100
TCP	363 ± 3	54
Bioglass	0	100
**Day 5**		
PCL	360 ± 2	54
HA	0	100
TCP	364 ± 1	54
Bioglass	0	100

**Table 4:** Pore size and porosity values of scaffolds (500 μm pore size) before and after the degradation process.

Materials	Pore Size (μm)	Porosity (%)
**Day 0**		
PCL	502 ± 3	68
HA	506 ± 4	68
TCP	510 ± 2	68
Bioglass	508 ± 4	68
**Day 1**		
PCL	508 ± 5	68
HA	608 ± 1	70
TCP	515 ± 6	68
Bioglass	544 ± 3	69
**Day 2**		
PCL	538 ± 6	68
HA	616 ± 5	70
TCP	552 ± 4	69
Bioglass	617 ± 4	70
**Day 3**		
PCL	550 ± 5	69
HA	837 ± 4	84
TCP	561 ± 5	69
Bioglass	0	100
**Day 4**		
PCL	556 ± 6	69
HA	0	100
TCP	570 ± 7	70
Bioglass	0	100
**Day 5**		
PCL	568 ± 8	69
HA	0	100
TCP	583 ± 1	70
Bioglass	0	100

**Table 5:** Designed and printed concentrations of PCL, HA, TCP and bioglass on the bone bricks and corresponding variations at different degradation time points.

Bone Bricks	Designed Concentration of Inorganic Material (wt%)	Measured Concentration of Inorganic Material (wt%)	Inorganic Material that Remained (wt%)	Degradation Temperature (°C)
**Day 0**				
PCL	0	0	0	437.11 ± 0.21
HA	20	20.27 ± 0.001	100	425.55 ± 0.38
TCP	20	20.38 ± 0.05	100	424.04 ± 0.23
Bioglass	20	20.68 ± 0.05	100	412.67 ± 0.18
**Day 1**				
PCL	-	0	0	436.95 ± 1.07
HA	-	21.64 ± 0.54	19.26	392.71 ± 1.42
TCP	-	19.45 ± 0.43	20.14	414.27 ± 0.18
Bioglass	-	20.73 ± 0.04	19.45	343.03 ± 0.12
**Day 2**				
PCL	-	0	0	435.79 ± 4.43
HA	-	20.77 ± 0.71	16.08	389.37 ± 2.51
TCP	-	19.41 ± 0.41	19.59	413.97 ± 0.86
Bioglass	-	21.71 ± 0.32	11.57	325.56 ± 2.26
**Day 3**				
PCL	-	0	0	431.83 ± 2.67
HA	-	22.44 ± 1.4	9.44	381.22 ± 2.63
TCP	-	18.92 ± 0.44	18.92	411.85 ± 0.12
Bioglass	-	22.14 ± 0.76	7.14	317.15 ± 2.16
**Day 4**				
PCL	-	0	0	431.83 ± 2.67
HA	-	21.11 ± 0.55	5.27	372.81 ± 1.64
TCP	-	18.69 ± 0.14	18.55	410.1 ± 1.21
Bioglass	-	18.12 ± 0.01	4.75	312.83 ± 3.41
**Day 5**				
PCL	-	0	0	427.87 ± 0.13
HA	-	21.34 ± 0.04	3.77	367.33 ± 9.5
TCP	-	18.17 ± 0.22	18.17	409.93 ± 0.01
Bioglass	-	19.14 ± 0.25	2.01	304.12 ± 5.78

**Table 6:** Key thermal properties and crystallinity values for all samples at different degradation time points.

Material	Day	Tg (°C)	Tm (°C)	ΔHm (J/g)	χ_c_ (%)
PCL	Day 0	−59.19	64.65	89.89	86.91
	Day 1	−58.09	61.48	83.92	81.13
	Day 2	−58.03	60.83	81.23	78.54
	Day 3	−57.64	60.29	80.69	78.02
	Day 4	−55.81	55.06	79.2	76.58
	Day 5	−54.46	54.85	74.67	72.2
HA	Day 0	−59.22	60.98	65.06	61.08
	Day 1	−57.76	59.19	64.98	61.86
	Day 2	−56.74	58.85	64.98	51.98
	Day 3	−57	58	64.21	55.12
	Day 4	−56.45	57.63	63.92	15.94
	Day 5	0	0	0	0
TCP	Day 0	−59.11	59.82	66.28	64.08
	Day 1	−58.58	59.73	66.16	63.96
	Day 2	−56.64	58.03	65.64	62.97
	Day 3	−58.57	57.8	64.58	60.82
	Day 4	−58.47	57.53	65.32	60.53
	Day 5	−58.41	57.42	65.22	59.37
Bioglass	Day 0	−59.1	59.94	68.48	66.21
	Day 1	−58.36	59.27	67.97	65.72
	Day 2	−57.82	57.33	64.34	48.4
	Day 3	−57.78	57.2	64.03	34.51
	Day 4	−57.69	56.01	63.91	25.76
	Day 5	−59.1	0	0	0

## Data Availability

Not applicable.

## Supplementary Materials

The following supporting information can be downloaded at: https://www.mdpi.com/article/10.3390/polym15030670/s1, Figure S1. Top and cross section images of PCL bone bricks at different degradation time points. (A,B) Day 1; (C,D) Day 2; (E,F), Day 3; (G,H) Day 4; (I,J) Day 5; Figure S2. Top and cross section images of PCL/HA bone bricks at different degradation time points. (A,B) Day 1; (C,D) Day 2; (E,F), Day 3; (G,H) Day 4; (I.J) Day 5; Figure S3. Top and cross section images of PCL/TCP bone bricks at different degradation time points. (A,B) Day 1; (C,D) Day 2; (E,F), Day 3; (G,H) Day 4; (I,J) Day 5; Figure S4. Top and cross section images of PCL/bioglass bone bricks at different degradation time points. (A,B) Day 1; (C,D) Day 2; (E,F), Day 3; (G,H) Day 4; (I) Day 5; Figure S5. Top and cross section images of PCL rectangular scaffolds with 200 μm of pore size at different degradation time points. (A,B) Day 1; (C,D) Day 2; (E,F) Day 3; (G,H) Day 4; (I,J) Day 5; Figure S6. Top and cross section images of PCL rectangular scaffolds with 300 μm of pore size at different degradation time points. (A,B) Day 1; (C,D) Day 2; (E,F) Day 3; (G,H) Day 4; (I,J) Day 5; Figure S7. Top and cross section images of PCL rectangular scaffolds with 500 μm of pore size at different degradation time points. (A,B) Day 1; (C,D) Day 2; (E,F) Day 3; (G,H) Day 4; (I,J) Day 5; Figure S8. Top and cross section images of PCL/HA rectangular scaffolds with 200 μm of pore size at different degradation time points. (A,B) Day 1; (C,D) Day 2; (E) Day 3; Figure S9. Top and cross section images of PCL/HA rectangular scaffolds with 300 μm of pore size at different degradation time points. (A,B) Day 1; (C,D) Day 2; (E) Day 3; Figure S10. Top and cross section images of PCL/HA rectangular scaffolds with 500 μm of pore size at different degradation time points. (A,B) Day 1; (C,D) Day 2; Figure S11. Top and cross section images of PCL/TCP rectangular scaffolds with 200 μm of pore size at different degradation time points. (A,B) Day 1; (C,D) Day 2; (E,F) Day 3; (G,H) Day 4; (I,J) Day 5; Figure S12. Top and cross section images of PCL/TCP rectangular scaffolds with 300 μm of pore size at different degradation time points. (A,B) Day 1; (C,D) Day 2; (E,F) Day 3; (G,H) Day 4; (I,J) Day 5; Figure S13. Top and cross section images of PCL/TCP rectangular scaffolds with 500 μm of pore size at different degradation time points. (A,B) Day 1; (C,D) Day 2; (E,F) Day 3; (G,H) Day 4; (I,J) Day 5; Figure S14. Top and cross section images of PCL/bioglass rectangular scaffolds with 200 μm of pore size at different degradation time points. (A,B) Day 1; (C,D) Day 2; Figure S15. Top and cross section images of PCL/bioglass rectangular scaffolds with 300 μm of pore size at different degradation time points. (A,B) Day 1; (C) Day 2; Figure S16. Thermal Gravimetric Analysis (TGA) curves of bone bricks at different degradation times. (A) Day 1, (B) Day 2, (C) Day 3, (D) Day 4 and (E) Day 5; Figure S17. Thermal Gravimetric Analysis (TGA) curves of bone bricks samples as a function of degradation time for different material compositions. (A) PCL, (B) PCL/HA, (C) PCL/TCP and (D) PCL/bioglass; Figure S18. Glass transition temperature and heating curves for bone bricks containing different material composition as a function of degradation time. (A) Glass transition temperature on day 0, (B) Heating curve on day 0, (C) Glass transition temperature on day 1, (D) Heating curve on day 1, (E) Glass transition temperature on day 2 and (F) Heating curve on day 2, (G) Glass transition temperature on day 3, (H) Heating curve on day 3, (I) Glass transition temperature on day 4, (J) Heating curve on day 4, (K) Glass transition temperature on day 5 and (L) Heating curve on day 5.
