# Supplementary material for: Accelerated Degradation of Poly-ε-caprolactone Composite Scaffolds for Large Bone Defects
Source: Polymers (Basel). 2023 Jan 28;15(3):670. doi: 10.3390/polym15030670 (PMC9921763; doi:10.3390/polym15030670)
Supplement: Supplementary file 1 [file polymers-15-00670-s001.zip › polymers-2106534-SM.pdf]

polymers

Supplementary Materials

Accelerated Degradation of Poly-ε-Caprolactone Composite Scaffolds for Large Bone Defects

Evangelos Daskalakis [1,*], Mohamed H. Hassan [1], Abdalla M. Omar [1], Anil A. Acar [2,3,4], Ali Fallah [2,3,4], Glen Cooper [1], Andrew Weightman [1], Gordon Blunn [5], Bahattin Koc [2,3,4] and Paulo Bartolo [1,6,*]

[1] School of Mechanical, Aerospace and Civil Engineering, University of Manchester, Manchester M13 9PL, UK; mohamed.hassan@manchester.ac.uk (M.H.H.); abdalla.omar@manchester.ac.uk (A.M.O.); glen.cooper@manchester.ac.uk (G.C.); andrew.weightman@manchester.ac.uk (A.W.)
[2] Integrated Manufacturing Technologies Research and Application Center, Sabanci University, Tuzla 34956, Istanbul, Turkey; aacar@sabanciuniv.edu (A.A.A.); ali.fallah@sabanciuniv.edu (A.F.); bahattinkoc@sabanciuniv.edu (B.K.)
[3] SUNUM Nanotechnology Research Center, Sabanci University, Tuzla 34956, Istanbul, Turkey
[4] Faculty of Engineering and Natural Sciences, Sabanci University, Tuzla 34956, Istanbul, Turkey
[5] School of Pharmacy and Biomedical Sciences, University of Portsmouth, Portsmouth PO1 2DT, UK; gordon.blunn@port.ac.uk
[6] Singapore Centre for 3D Printing, School of Mechanical and Aerospace Engineering, Nanyang Technological University, Singapore 639798, Singapore
* Correspondence: evangelos.daskalakis@manchester.ac.uk (E.D.); pbartolo@ntu.edu.sg (P.B.)

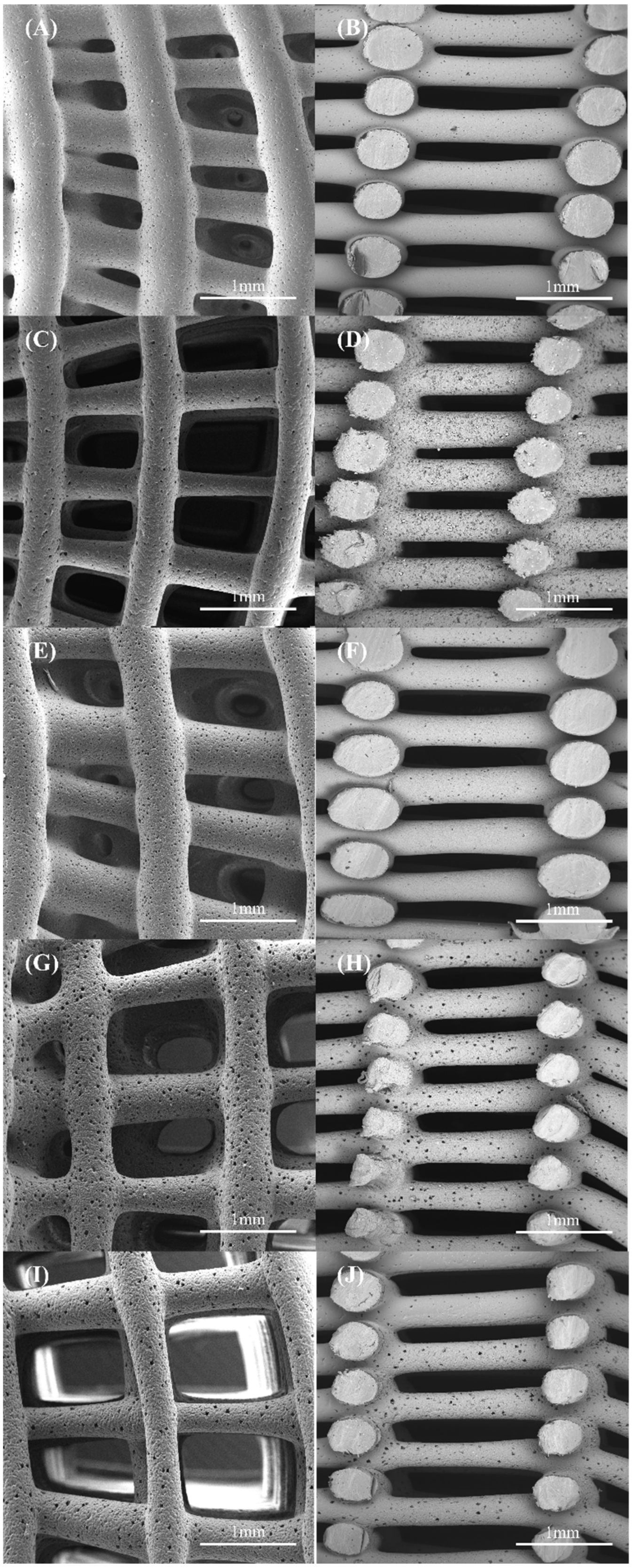

Figure S1. Top and cross section images of PCL bone bricks at different degradation time points. (A,B) Day 1; (C,D) Day 2; (E,F), Day 3; (G,H) Day 4; (I,J) Day 5.

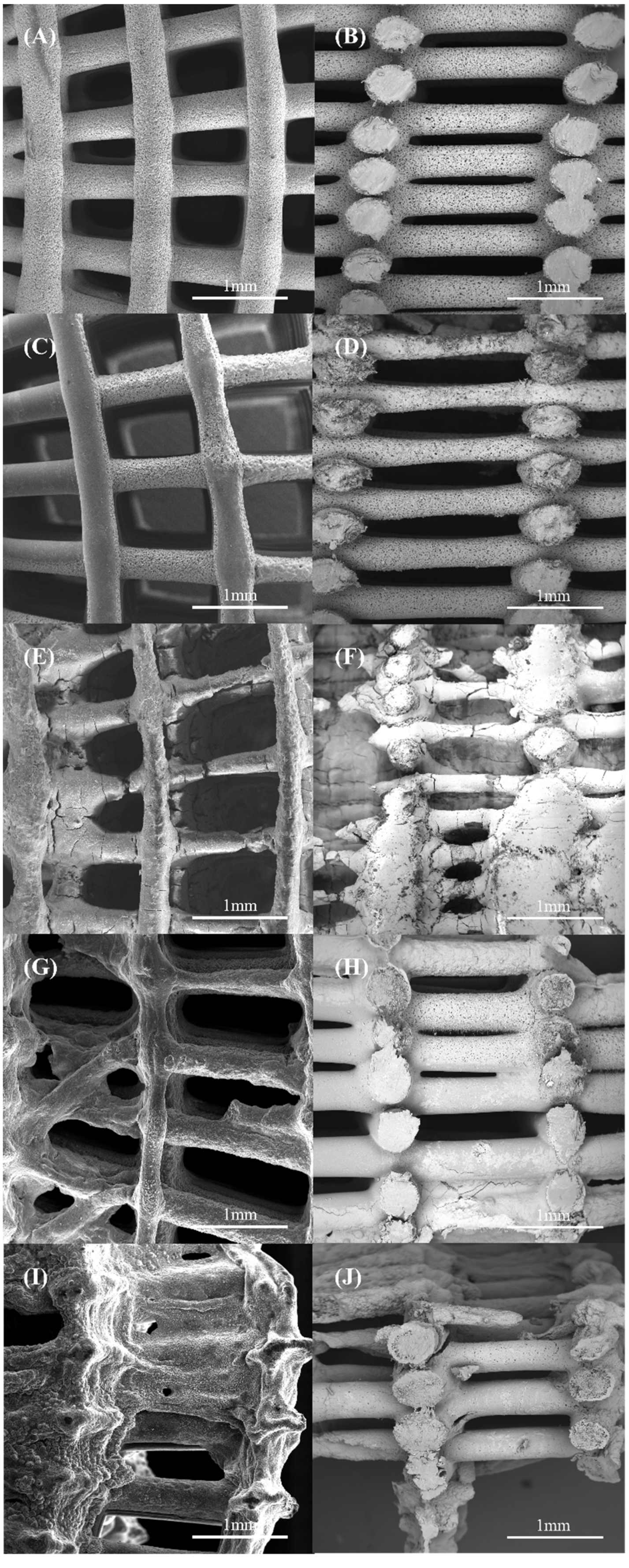

Figure S2. Top and cross section images of PCL/HA bone bricks at different degradation time points. (A,B) Day 1; (C,D) Day 2; (E,F), Day 3; (G,H) Day 4; (I,J) Day 5.

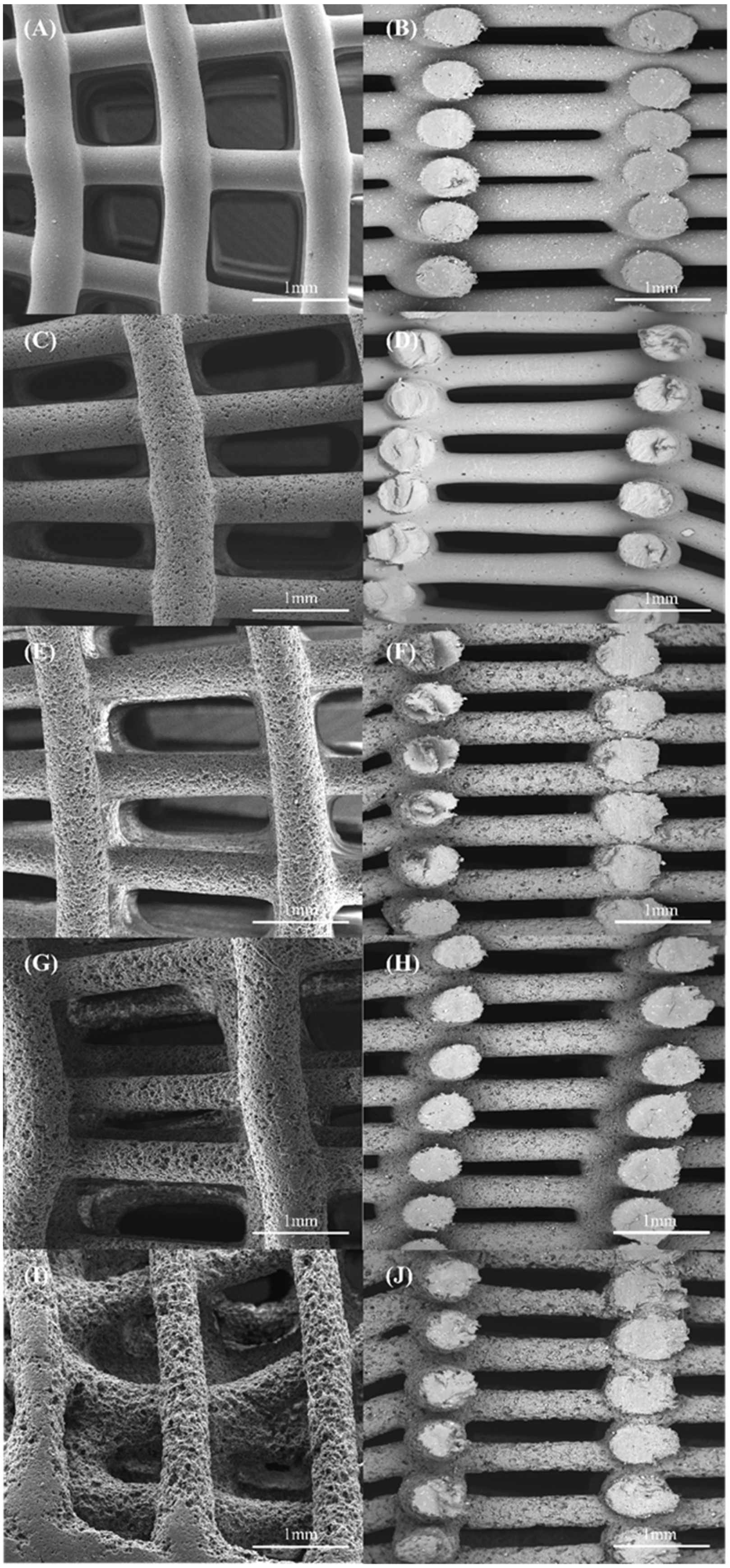

Figure S3. Top and cross section images of PCL/TCP bone bricks at different degradation time points. (A,B) Day 1; (C,D) Day 2; (E,F), Day 3; (G,H) Day 4; (I,J) Day 5.

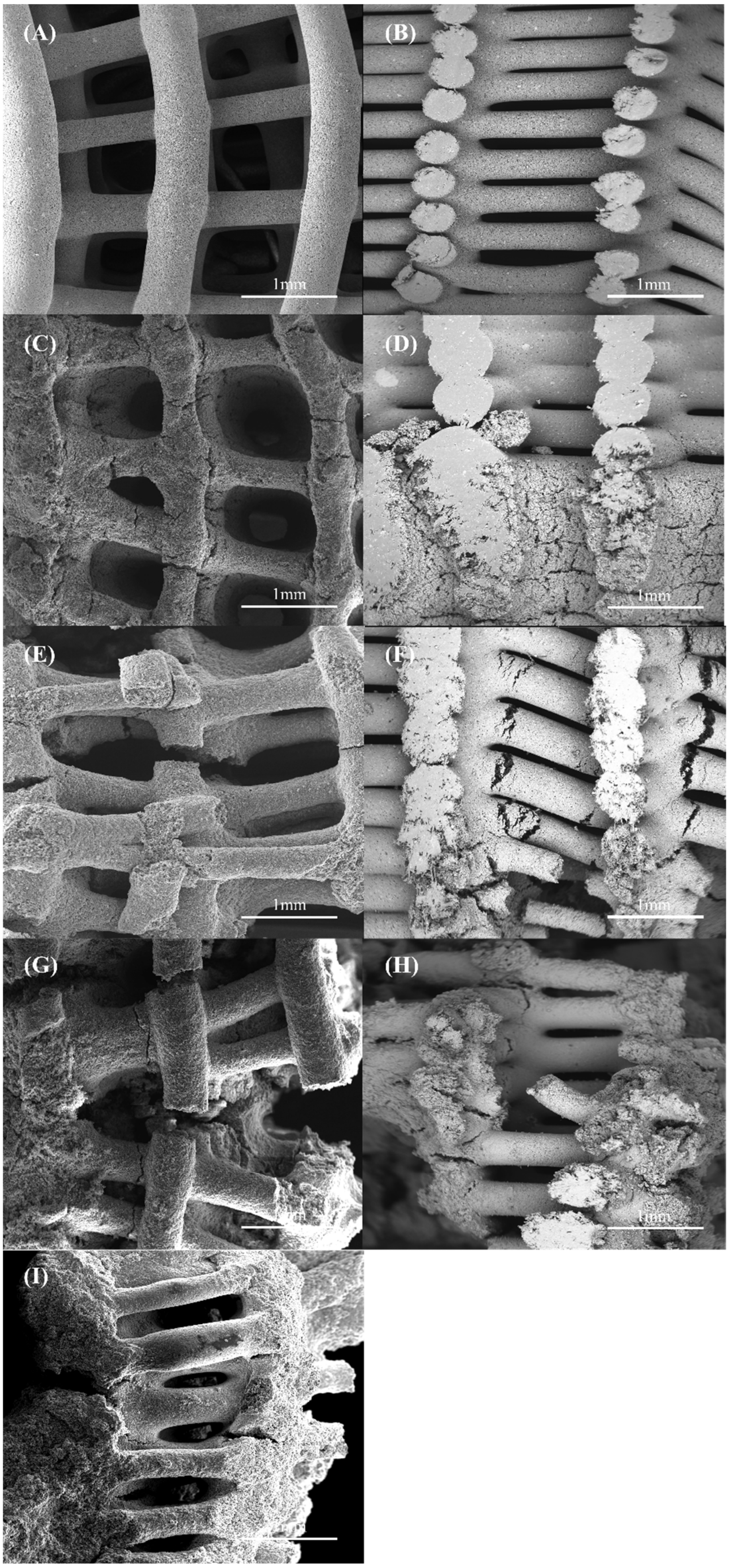

Figure S4. Top and cross section images of PCL/bioglass bone bricks at different degradation time points. (A,B) Day 1; (C,D) Day 2; (E,F), Day 3; (G,H) Day 4; (I) Day 5.

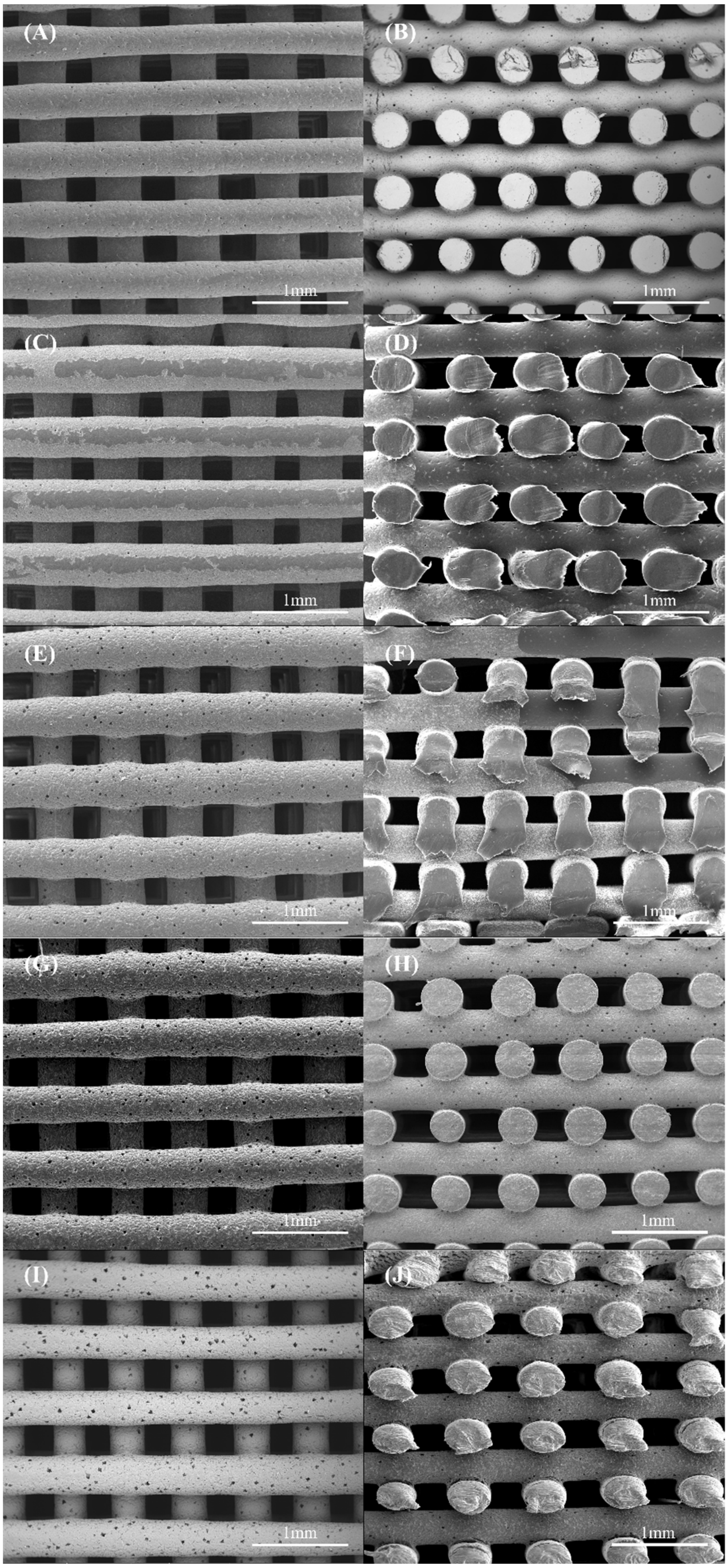

Figure S5. Top and cross section images of PCL rectangular scaffolds with 200 μm of pore size at different degradation time points. (A,B) Day 1; (C,D) Day 2; (E,F) Day 3; (G,H) Day 4; (I,J) Day 5.

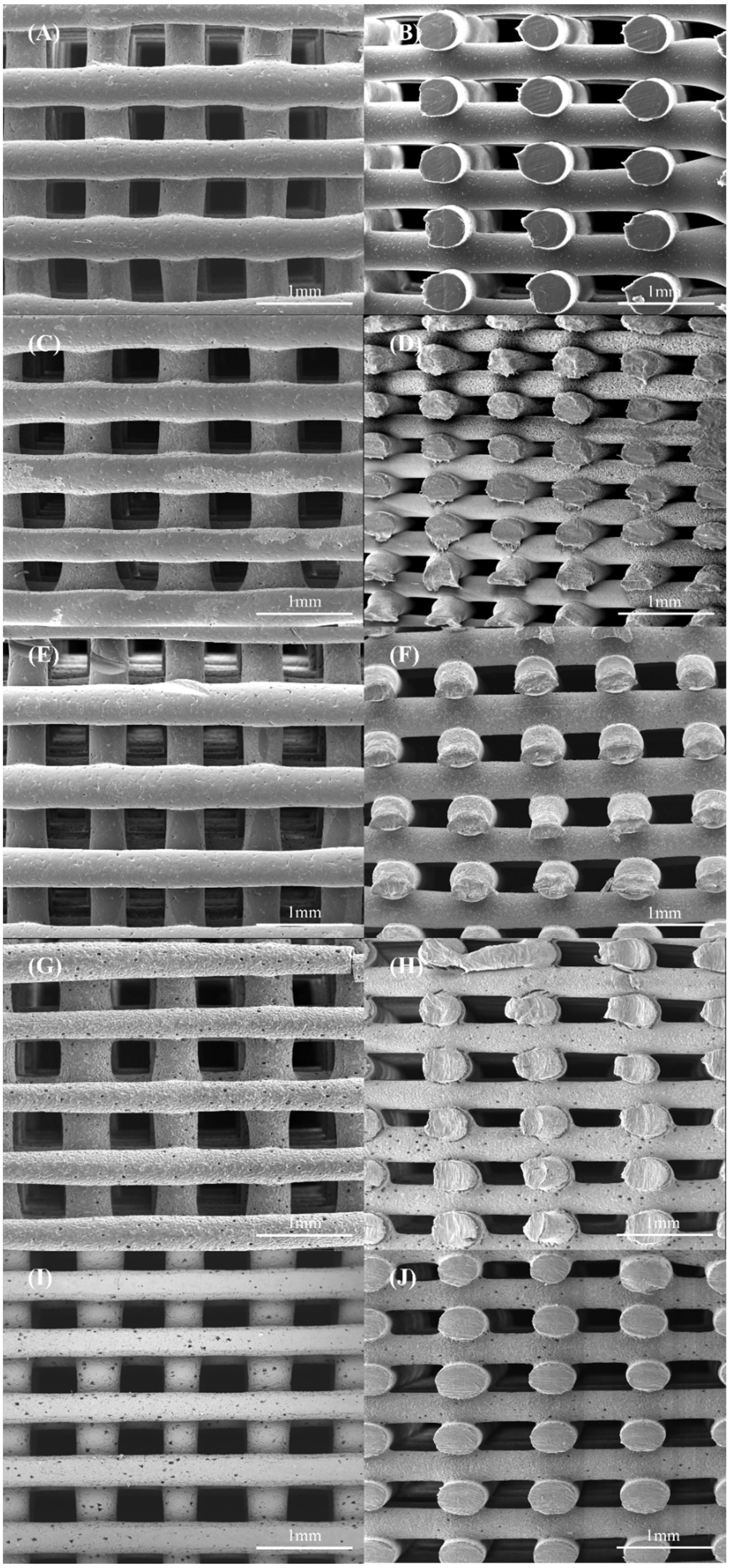

Figure S6. Top and cross section images of PCL rectangular scaffolds with 300 μm of pore size at different degradation time points. (A,B) Day 1; (C,D) Day 2; (E,F) Day 3; (G,H) Day 4; (I,J) Day 5.

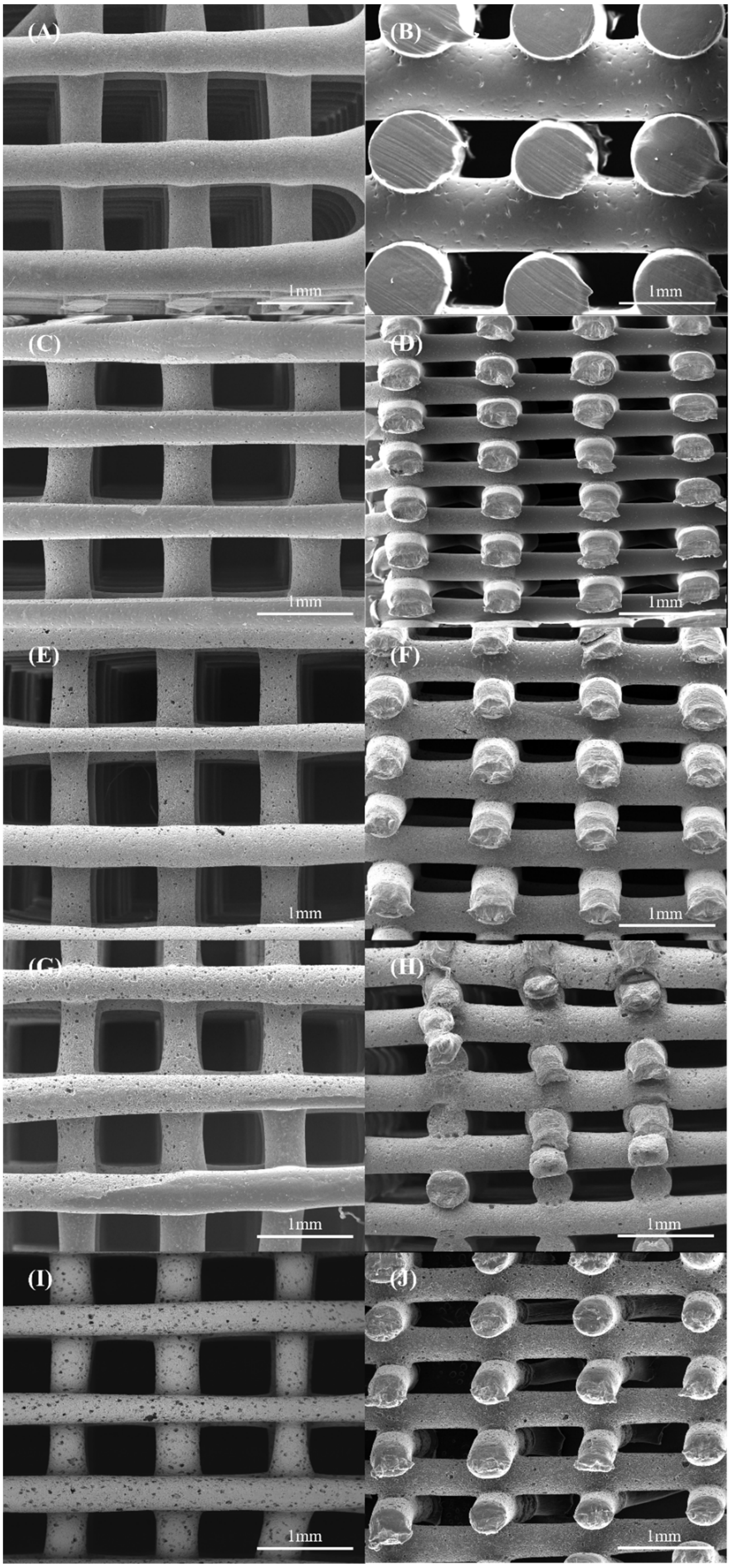

Figure S7. Top and cross section images of PCL rectangular scaffolds with 500 μm of pore size at different degradation time points. (A,B) Day 1; (C,D) Day 2; (E,F) Day 3; (G,H) Day 4; (I,J) Day 5.

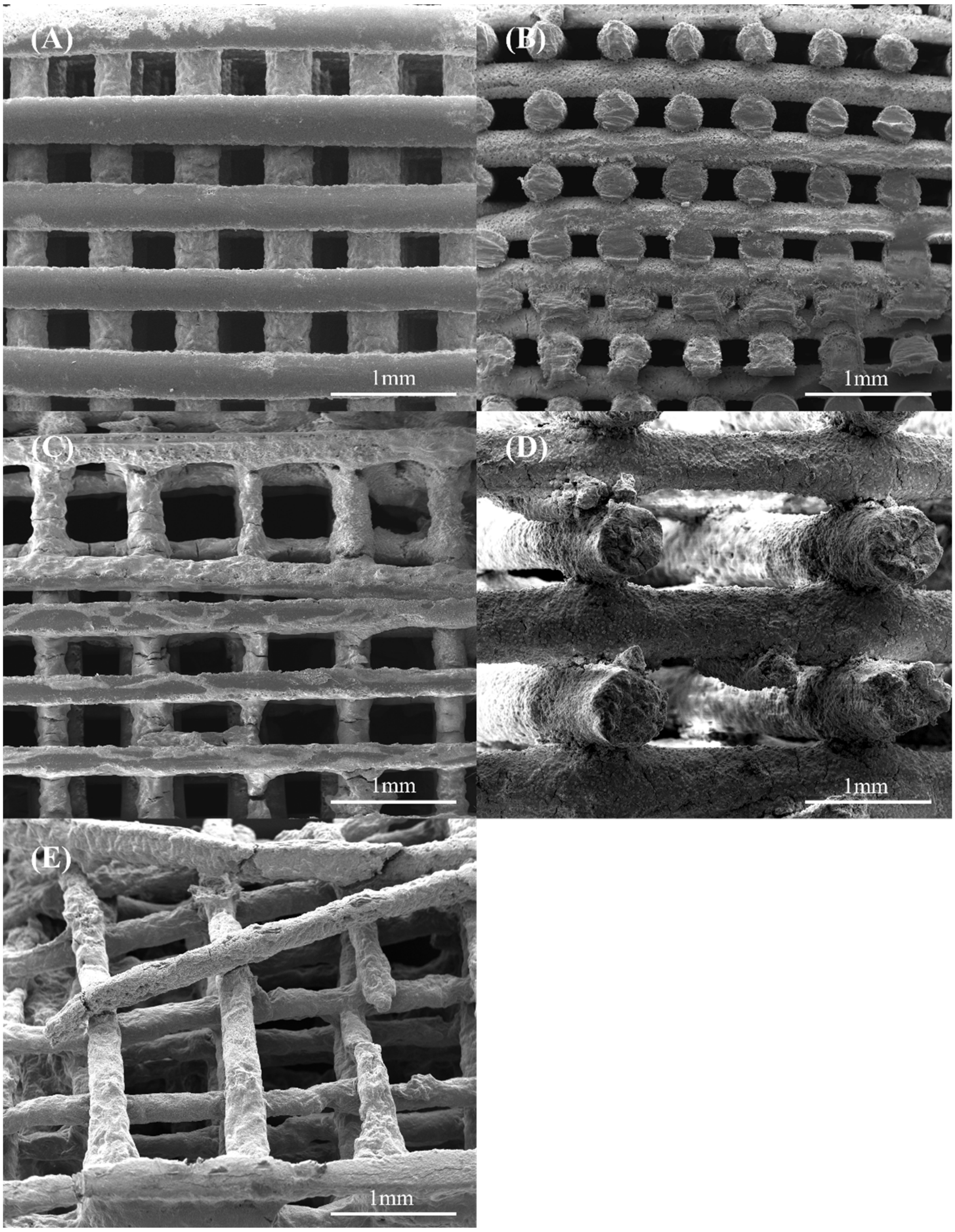

Figure S8. Top and cross section images of PCL/HA rectangular scaffolds with 200 µm of pore size at different degradation time points. (A,B) Day 1; (C,D) Day 2; (E) Day 3.

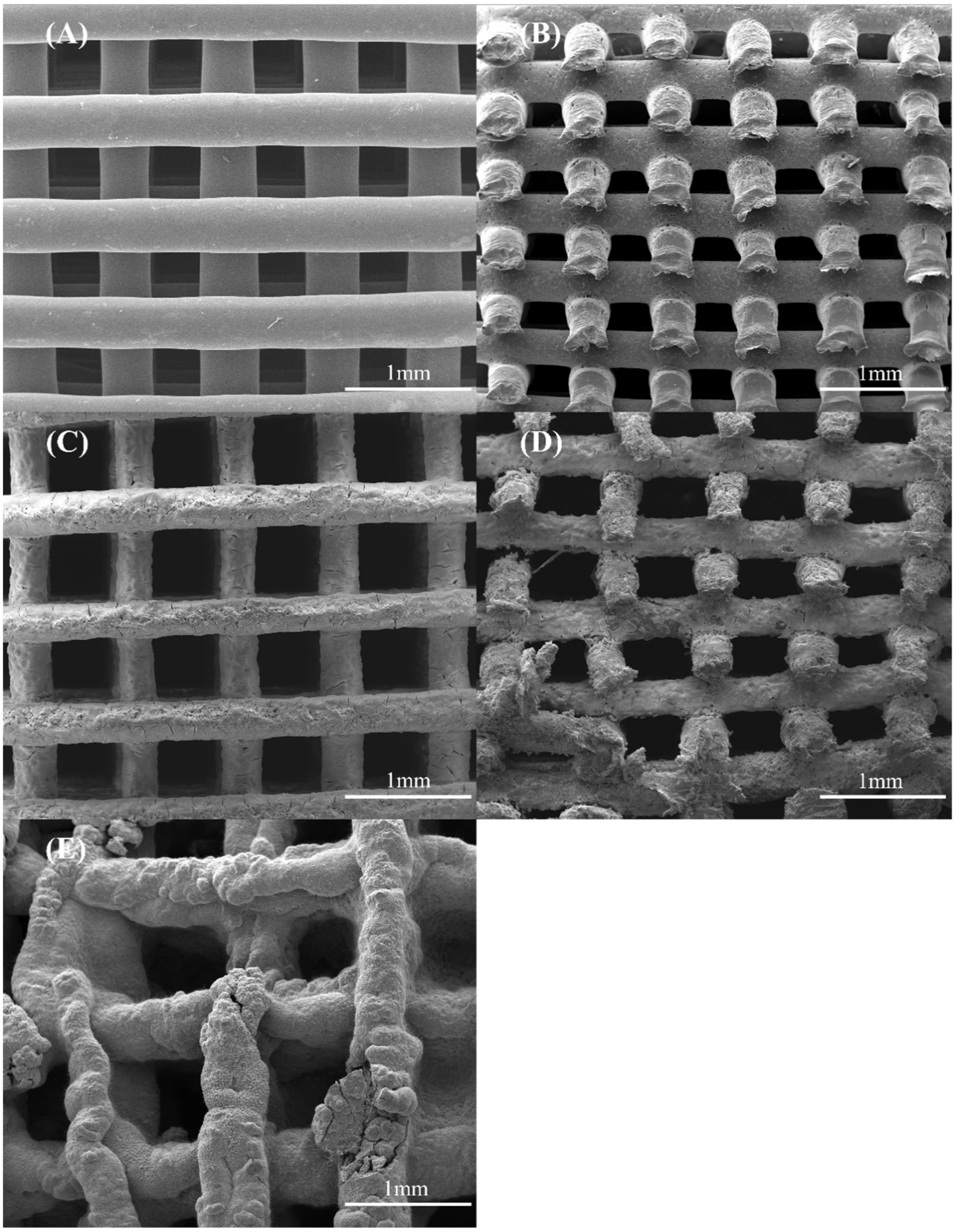

Figure S9. Top and cross section images of PCL/HA rectangular scaffolds with 300 μm of pore size at different degradation time points. (A,B) Day 1; (C,D) Day 2; (E) Day 3.

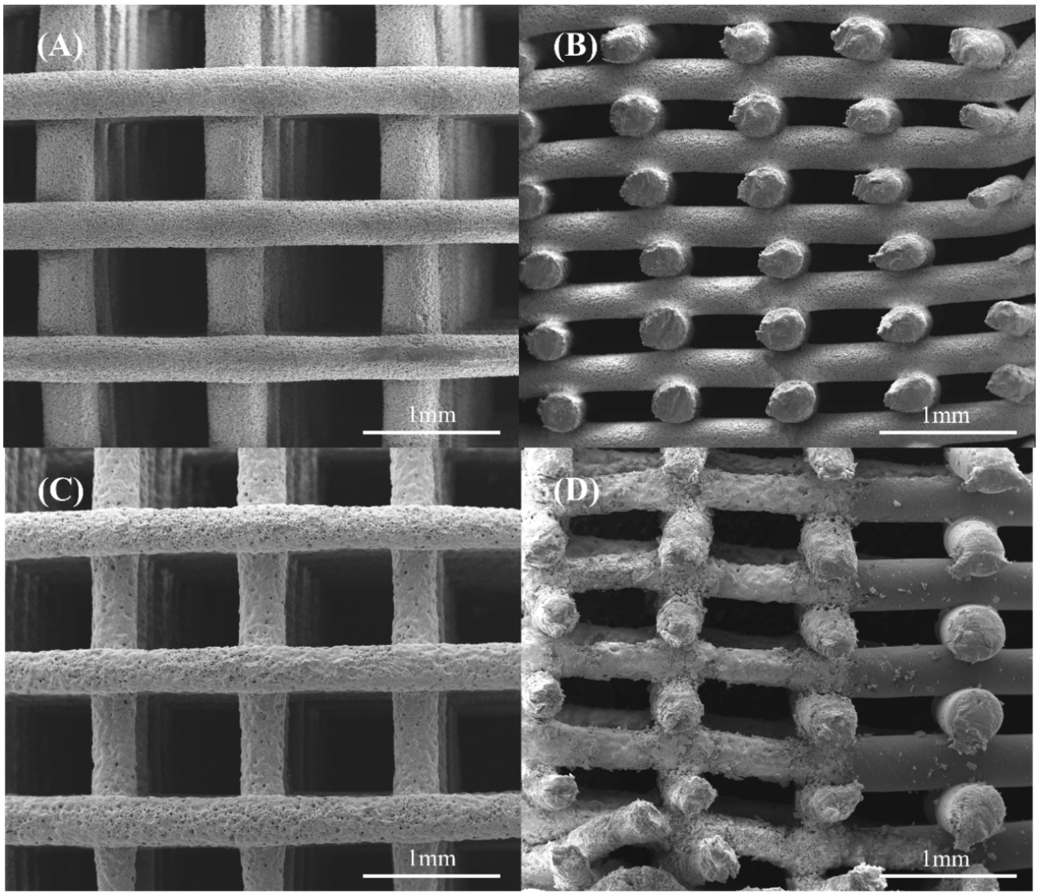

Figure S10. Top and cross section images of PCL/HA rectangular scaffolds with 500 μm of pore size at different degradation time points. (A,B) Day 1; (C,D) Day 2.

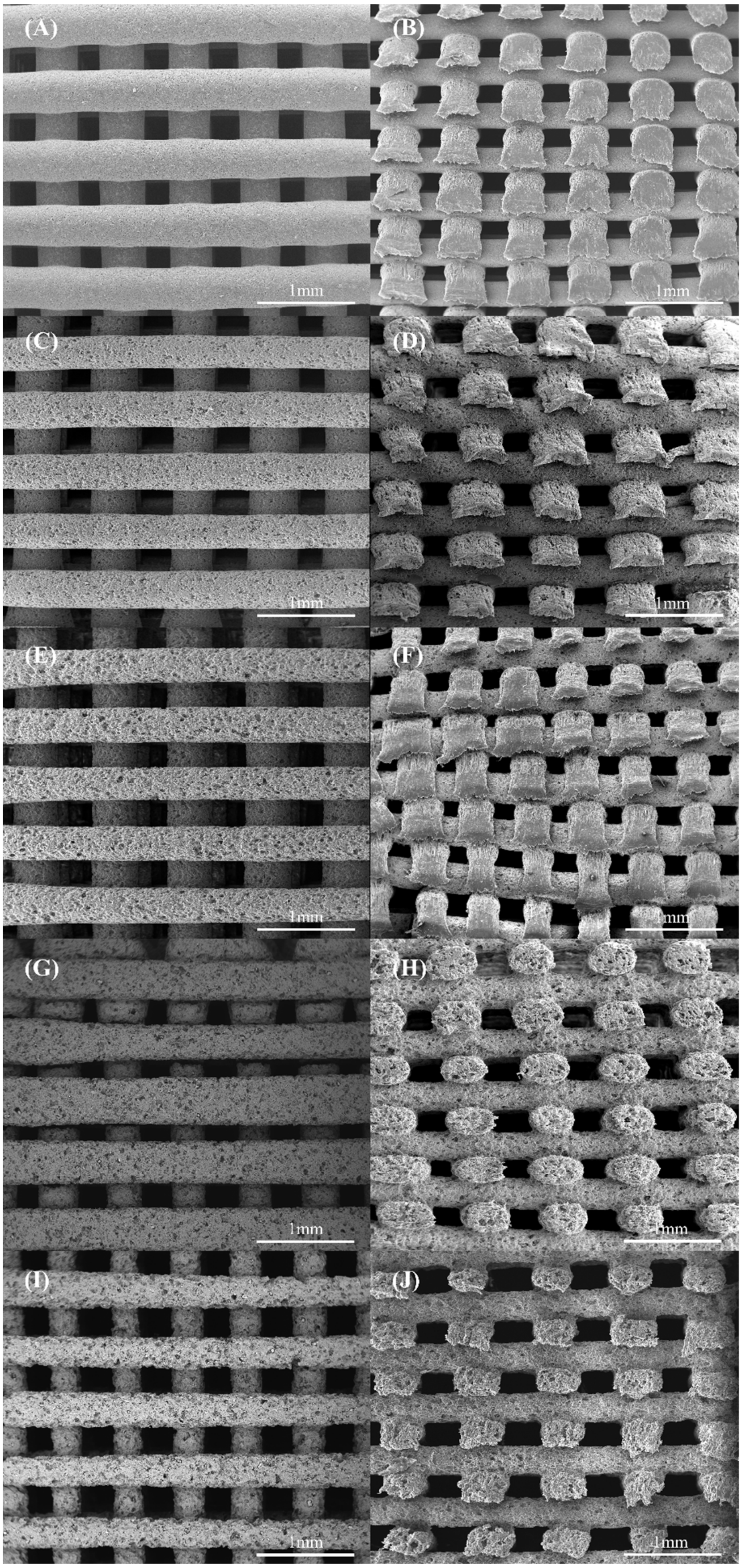

Figure S11. Top and cross section images of PCL/TCP rectangular scaffolds with 200 μm of pore size at different degradation time points. (A,B) Day 1; (C,D) Day 2; (E,F) Day 3; (G,H) Day 4; (I,J) Day 5.

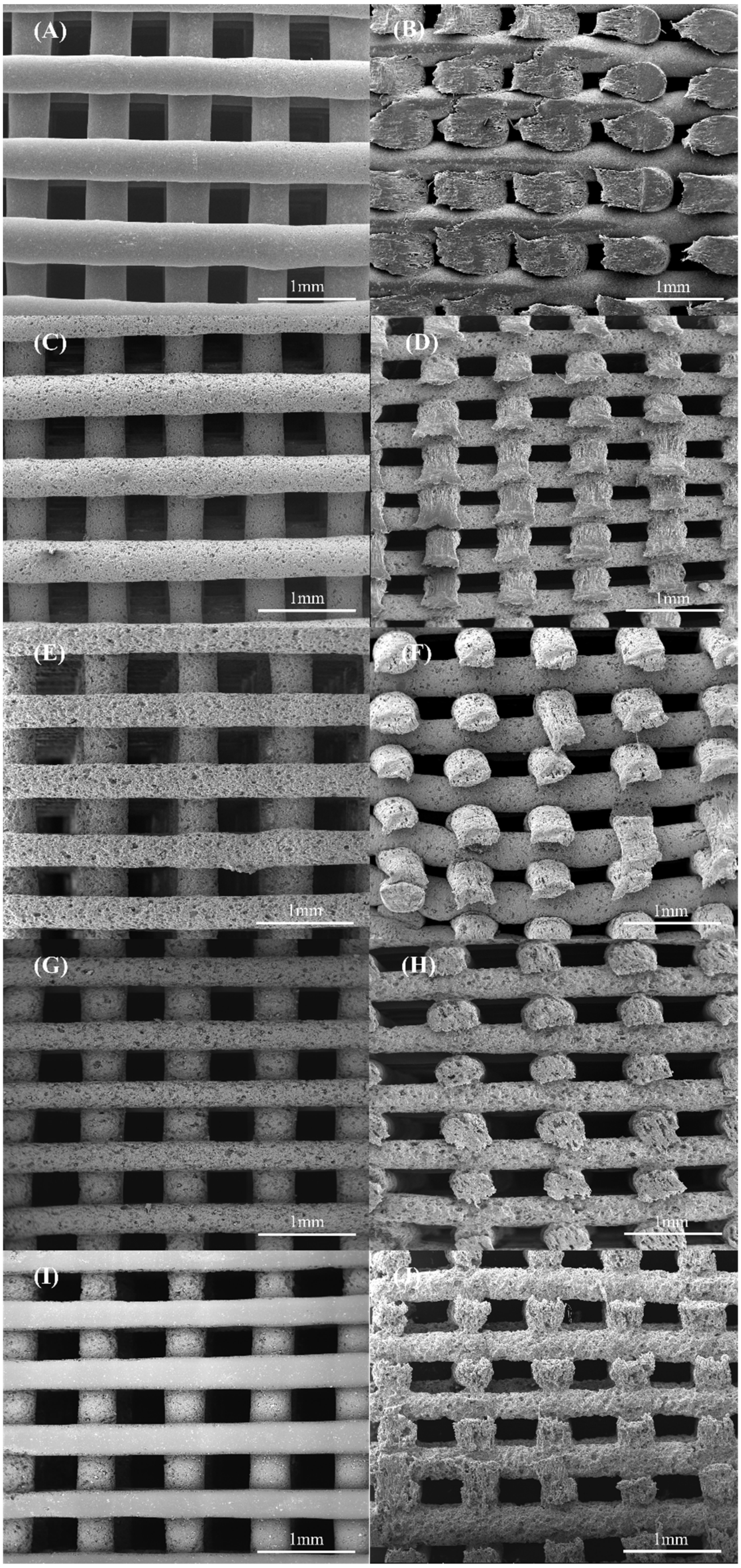

Figure S12. Top and cross section images of PCL/TCP rectangular scaffolds with 300 μm of pore size at different degradation time points. (A,B) Day 1; (C,D) Day 2; (E,F) Day 3; (G,H) Day 4; (I,J) Day 5.

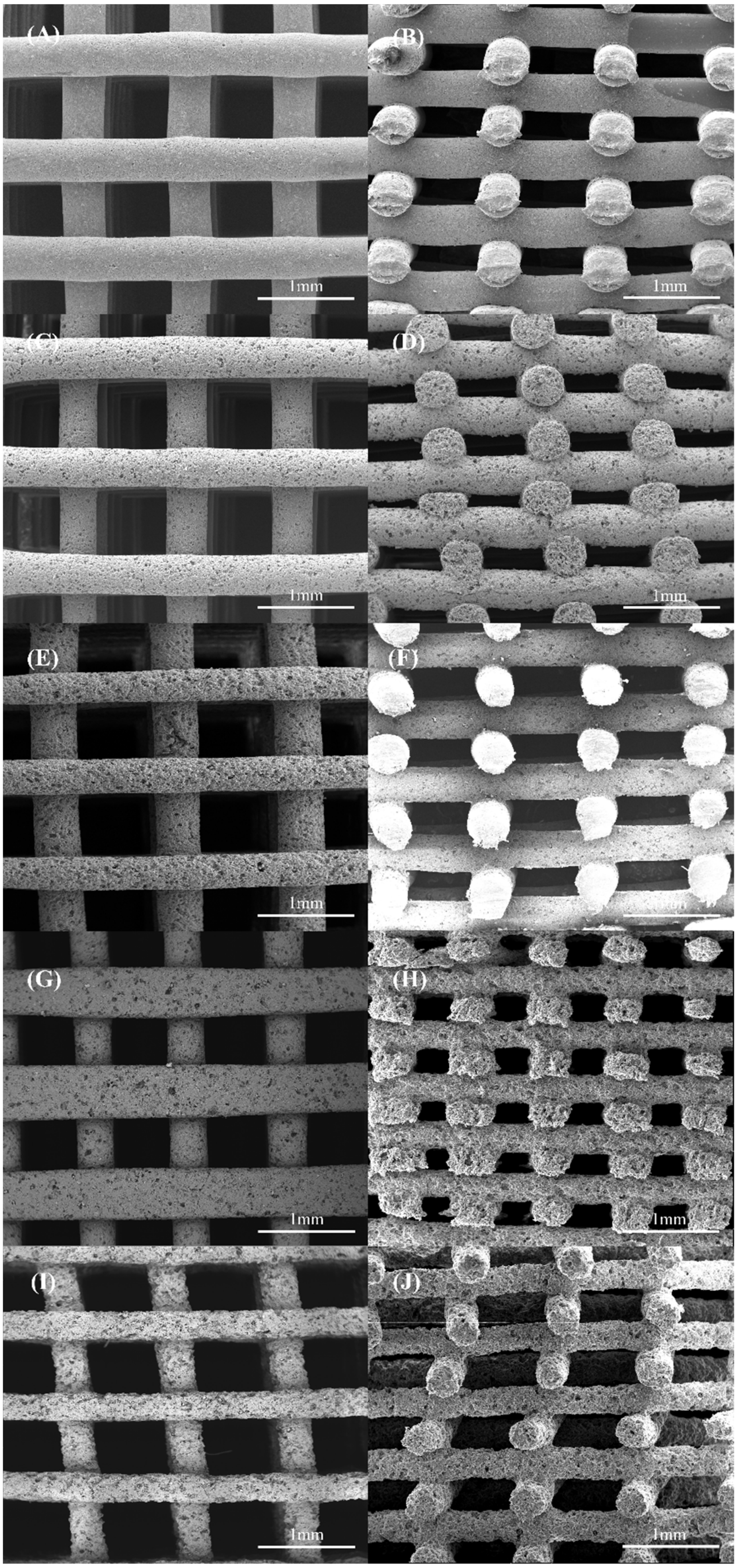

Figure S13. Top and cross section images of PCL/TCP rectangular scaffolds with 500 μm of pore size at different degradation time points. (A,B) Day 1; (C,D) Day 2; (E,F) Day 3; (G,H) Day 4; (I,J) Day 5.

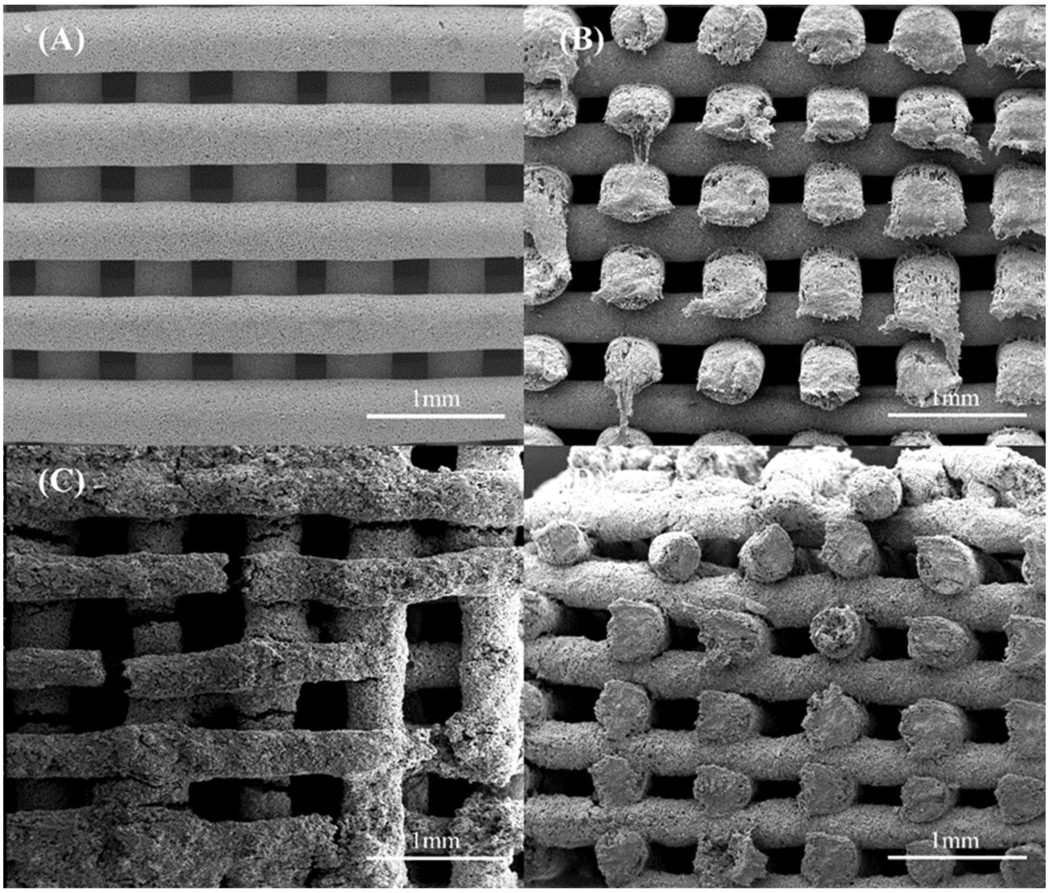

Figure S14. Top and cross section images of PCL/bioglass rectangular scaffolds with 200 μm of pore size at different degradation time points. (A,B) Day 1; (C,D) Day 2.

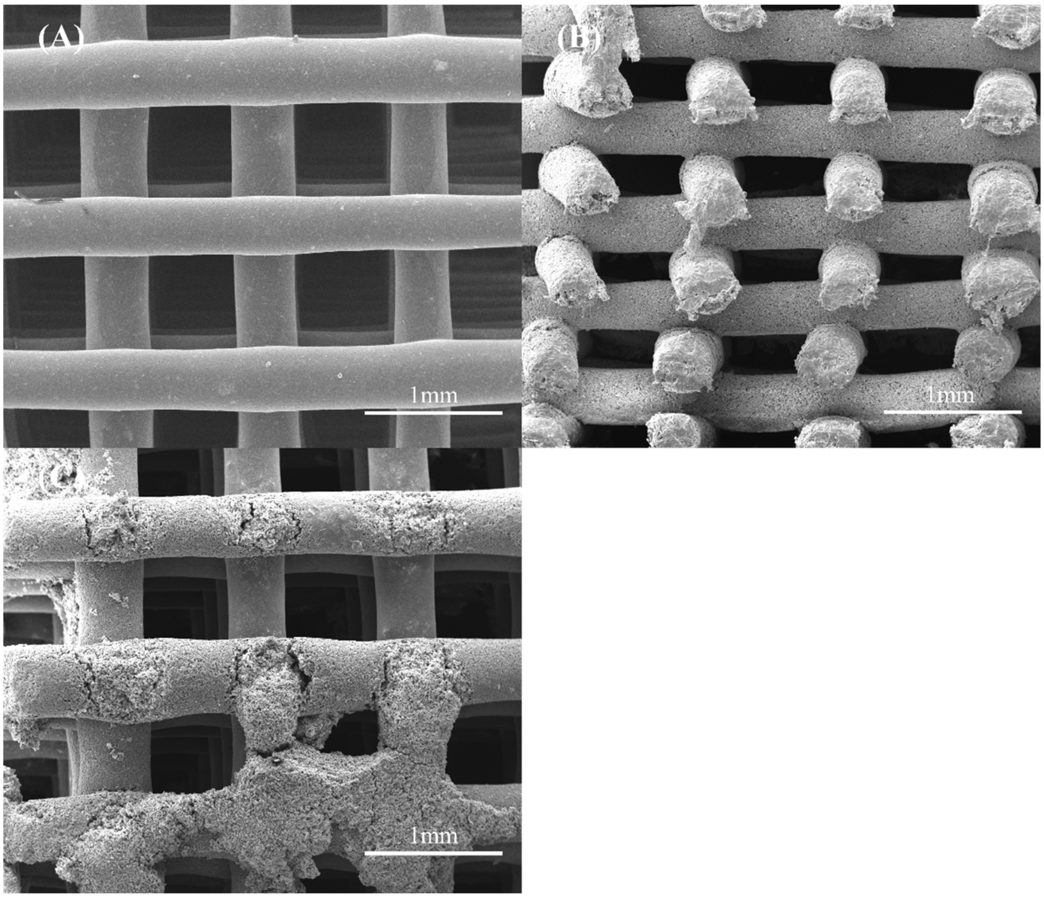

Figure S15. Top and cross section images of PCL/bioglass rectangular scaffolds with 300 μm of pore size at different degradation time points. (A,B) Day 1; (C) Day 2.

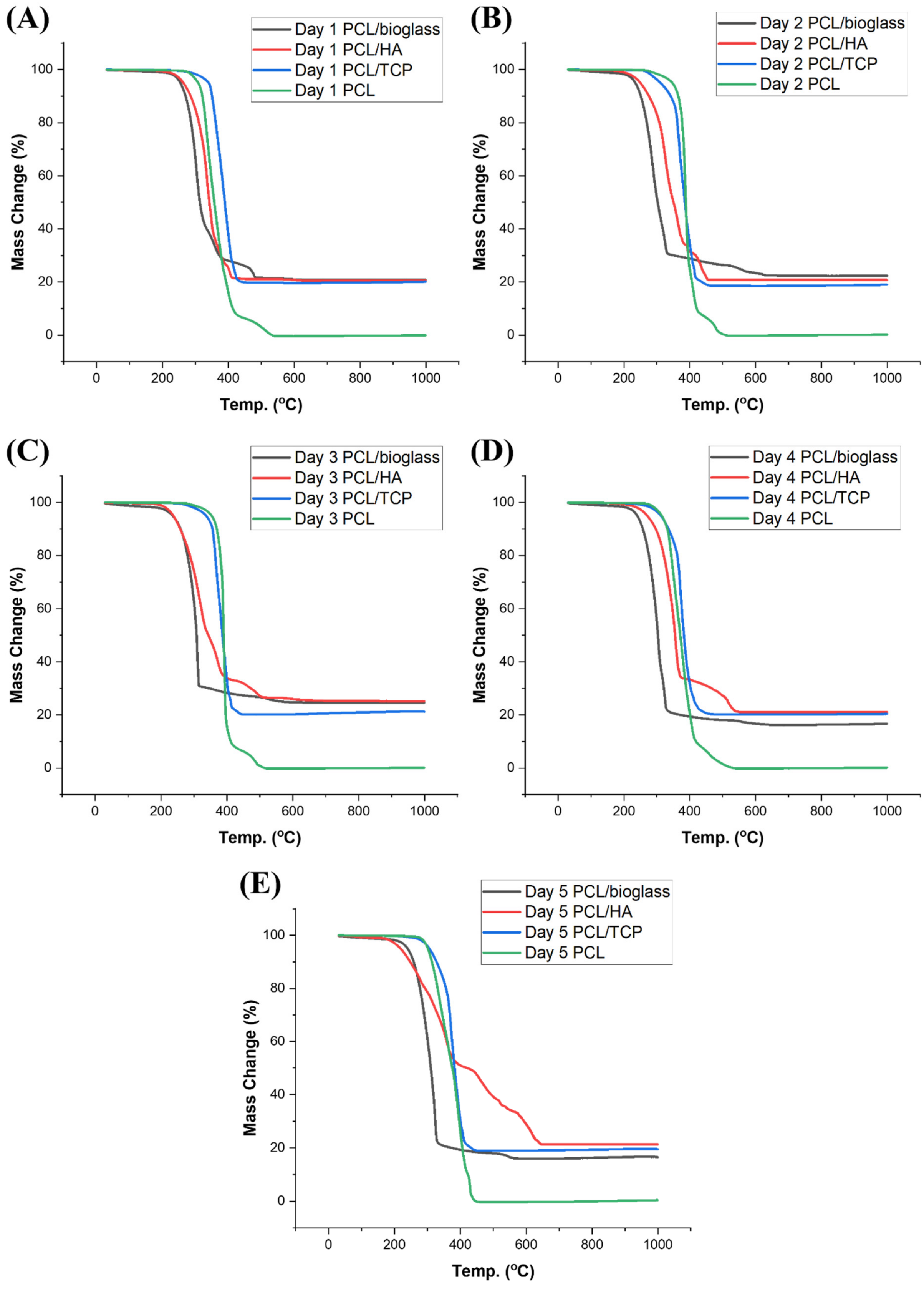

Figure S16. Thermal Gravimetric Analysis (TGA) curves of bone bricks at different degradation times. (A) Day 1, (B) Day 2, (C) Day 3, (D) Day 4 and (E) Day 5.

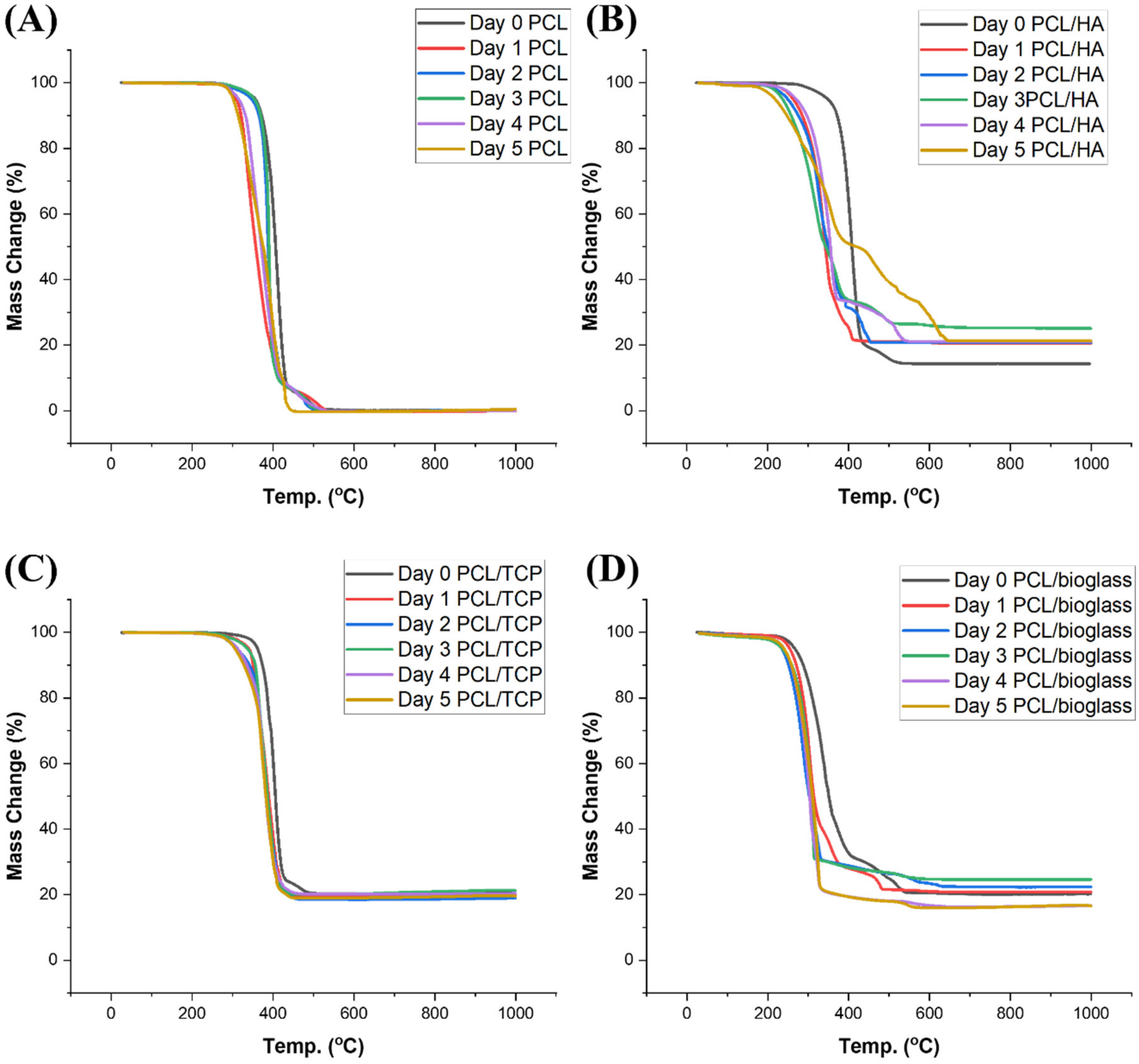

Figure S17. Thermal Gravimetric Analysis (TGA) curves of bone bricks samples as a function of degradation time for different material compositions. (A) PCL, (B) PCL/HA, (C) PCL/TCP and (D) PCL/bioglass.

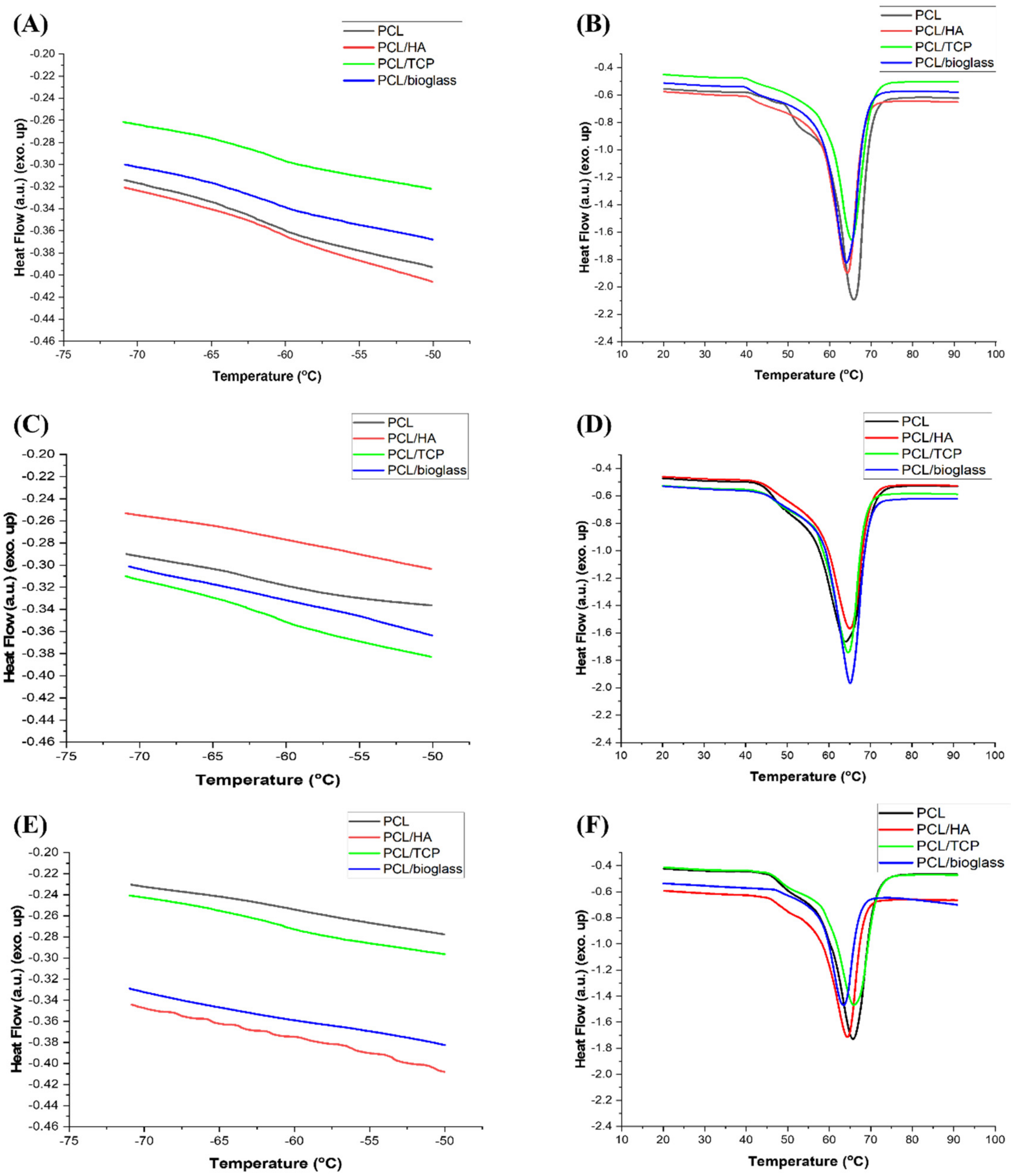

Figure S18. Glass transition temperature and heating curves for bone bricks containing different material composition as a function of degradation time. (A) Glass transition temperature on day 0, (B) Heating curve on day 0, (C) Glass transition temperature on day 1, (D) Heating curve on day 1, (E) Glass transition temperature on day 2 and (F) Heating curve on day 2, (G) Glass transition temperature on day 3, (H) Heating curve on day 3, (I) Glass transition temperature on day 4, (J) Heating curve on day 4, (K) Glass transition temperature on day 5 and (L) Heating curve on day 5.

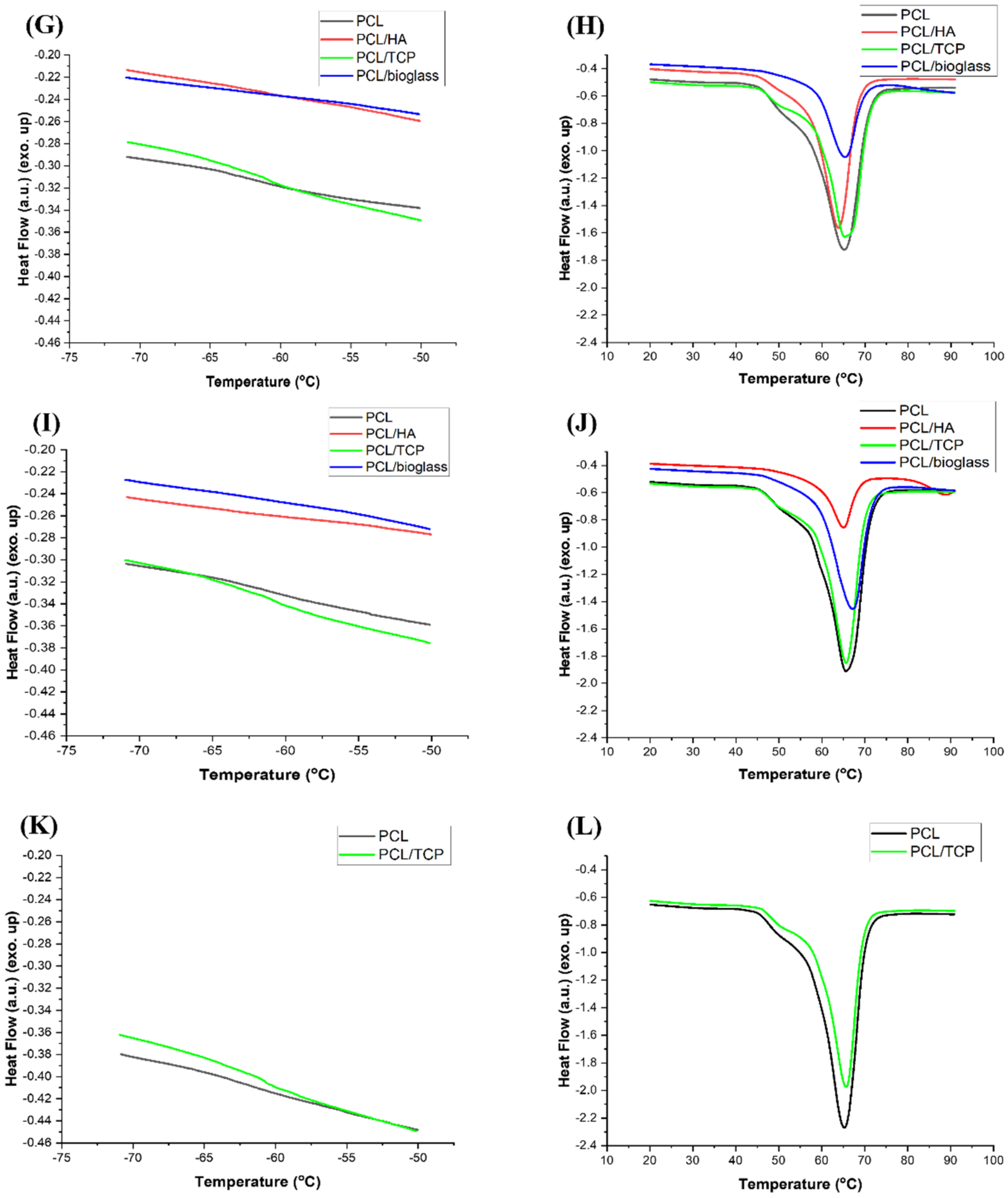

Figure S18. (cont.) Glass transition temperature and heating curves for bone bricks containing different material composition as a function of degradation time. (A) Glass transition temperature on day 0, (B) Heating curve on day 0, (C) Glass transition temperature on day 1, (D) Heating curve on day 1, (E) Glass transition temperature on day 2 and (F) Heating curve on day 2, (G) Glass transition temperature on day 3, (H) Heating curve on day 3, (I) Glass transition temperature on day 4, (J) Heating curve on day 4, (K) Glass transition temperature on day 5 and (L) Heating curve on day 5.
